# Striking global similarities in dog–human interactions

**DOI:** 10.1038/s41598-026-57657-1

**Published:** 2026-06-23

**Authors:** Juliane Bräuer, Yana Bender, Louise Jandke, Lea Ulverich, Henriette Mank, Christoph J. Völter, Lana Takau, Justorien Rambeloniaina, Roberto Zariquiey, Mariana Poblete, Stefan R. Schweinberger, Russell D. Gray

**Affiliations:** 1 DogStudies, Department for General Psychology and Cognitive Neuroscience, Friedrich Schiller University, Jena, Germany; 2 MPI GEA, Max Planck Institute of Geoanthropology, Jena, Germany; 3 Department of Comparative Cultural Psychology, Max Planck Institute for Evolutionary Anthropology, Leipzig, Germany; 4 Cognition and Applied Ethology, Messerli Research Institute, University of Veterinary Medicine Vienna, Medical University of Vienna and University of Vienna, Vienna, Austria; 5 Faculté de Médecine, Département d’Enseignement des Sciences et de Médecine Vétérinaires, Université d’Antananarivo, Antananarivo, Madagascar; 6 Chana Station for Language Sciences and Interculturality, Pontificia Universidad Católica del Perú, Lima, Peru; 7 Centre for Linguistics, Leiden University, Leiden, Netherlands; 8 Department of Linguistic and Cultural Evolution, Max Planck Institute for Evolutionary Anthropology, Leipzig, Germany; 9 School of Psychology, University of Auckland, Auckland, New Zealand

**Keywords:** Cultural differences, Domestication, Dog–human relationship, Animal cognition, Non-WEIRD countries, Ecology, Ecology, Evolution, Psychology, Psychology, Zoology

## Abstract

Most of our knowledge about the dog–human relationship comes from studies with dogs from ‘WEIRD’ (Western, Educated, Industrialized, Rich, and Democratic) societies. Here, we investigate cultural differences in dog–owner interactions worldwide. To achieve this, we developed a test battery comprising six well-established social-cognitive experiments and a questionnaire that assessed the psychological and practical aspects of the dog–human bond. We tested hunting dogs alongside their owners in five rural societies across culturally diverse locations in various countries: Vanuatu, Mongolia, Madagascar, Peru, and Germany. Despite dramatic cultural and environmental differences, we found that dog–human relationships were remarkably similar. Residual differences may be attributed to variations in hunting techniques and differences between WEIRD and non-WEIRD societies.

**Supplementary Information:**

The online version contains supplementary material available at 10.1038/s41598-026-57657-1.

## Introduction

Over the past 25 years, “human’s best friend” – domestic dogs (*Canis familiaris*) – have become a subject of increasing scientific study^1^. During the process of domestication, dogs have evolved human-like skills for functioning effectively in human societies^2–4^. In particular, they exhibit special skills in the social-communicative domain, allowing them to react appropriately to human gestures and language (e.g.^2,5,6^). The domestication process probably selected for dogs that were good cooperative partners^4,7–9^.

These special communicative and cooperative skills have been investigated in detail over the last two decades, including the dog’s use of the human pointing gesture, showing behavior, perspective-taking, social referencing, communication during an unsolvable problem, and obedience. It is well established that dogs use the human pointing gesture to locate hidden food, (although it is unclear to what extent the informative nature of the gesture^6,10–12^). In turn, dogs can also show a naive human the location of a hidden but non-accessible reward^13,14^. Furthermore, dogs avoid forbidden food that a human can see, indicating that they can take the visual perspective of the human^15^. Dogs also engage in social referencing, i.e. using the emotional information provided by humans about a novel, potentially dangerous object to guide their future behavior towards it^16,17^. When confronted with an unsolvable problem, dogs differ in their persistence; more importantly, they often look at a present human in that situation, potentially asking for help in that way^18,19^. Finally, in cooperative situations with humans, dogs follow the human’s lead^8^, and dog obedience plays a significant role in establishing a satisfying relationship between humans and dogs^20^.

However, nearly all of the above findings come from studies with dogs owned by people from Western, Educated, Industrialized, Rich, and Democratic (‘WEIRD’) societies^21^. But the majority of dogs in the world – approximately 75%- are not kept in the same manner as in Western countries^2,22^. Thus, the field of dog cognition suffers a similar problem to human psychology - typically, mainly WEIRD subjects are tested.

The absence of carefully planned cross-cultural studies on dog-owner dyads means we do not know whether dogs in non-Western cultures perform similarly to dogs from WEIRD countries in cognitive tests. In WEIRD countries the dog–human relationship is – among others – characterized by the following aspects: attachment^23^, effective communication (see above), increase in Oxytocin levels in both partners following socio-positive interactions^24,25^, and similarities in appearance and personality between dogs and their owners^26^. The question is whether these characteristics can be found worldwide, despite dramatic differences in culture and living conditions.

Two recent studies that have addressed the question of cultural differences in dog–human interactions in detail^27,28^ are based on ethnographies from cross-cultural databases. Chira et al. (2023) investigated dog–human relationships across 124 globally distributed societies^27^. The authors hypothesized that employing dogs for highly cooperative functions would lead to closer dog–human bonds. In particular, they expected increased positive care (e.g. dogs are allowed indoors, dogs receive healthcare, puppies are raised), decreased negative treatment (e.g. dogs are not fed, dogs are physically abused, dogs are regularly culled), and more attribution of personhood (e.g. dogs are named, dogs are buried and/or mourned, dogs are perceived as family members) in dogs with cooperative functions. Chira et al. (2023) found that hunting was the most frequent function in their huge sample, suggesting that it played a key role in dog domestication. Interestingly, they found increased odds of dog personhood in cultures that keep dogs for hunting^27^, see also^28^.

In addition to these studies based solely on ethnographic databases (which did not specifically focus on the human-dog relationship), there are a growing number of experimental studies with free-ranging dogs from non-WEIRD countries^29–31^. For example, Bhattacharjee and colleagues tested free-ranging dogs in India. They found that these dogs can follow distal pointing cues from humans to locate hidden food rewards similarly to dogs from Western societies^32^, see also^33,34^, suggesting that this is a universal cognitive skill among dogs. Bhattacharjee and colleagues also tested free-ranging dogs with the unsolvable problem, which were not persistent compared to WEIRD dogs and gazed at the human experimenter for longer durations^35^. Importantly, the subjects of these studies were free-ranging dogs, so these results can only contribute to understanding the universality of canine social cognition^30^, rather than dog–human bonds across cultures.

Thus, the primary objective of the current study was to investigate cultural differences in dog-owner interactions worldwide. To achieve this, we developed a test battery comprising well-established social-cognitive experiments and a questionnaire that assessed the psychological and practical aspects of the dog–human bond. We hypothesised that

1. there would be substantial cultural differences in dog–human relationships – 1a) between cultures and 1b) between Germany and non-WEIRD countries;
2. the dogs’ performance on the social cognitive tasks depends on the closeness of the dog-owner bond as indicated by the results of the questionnaire.

**Fig. 1:**
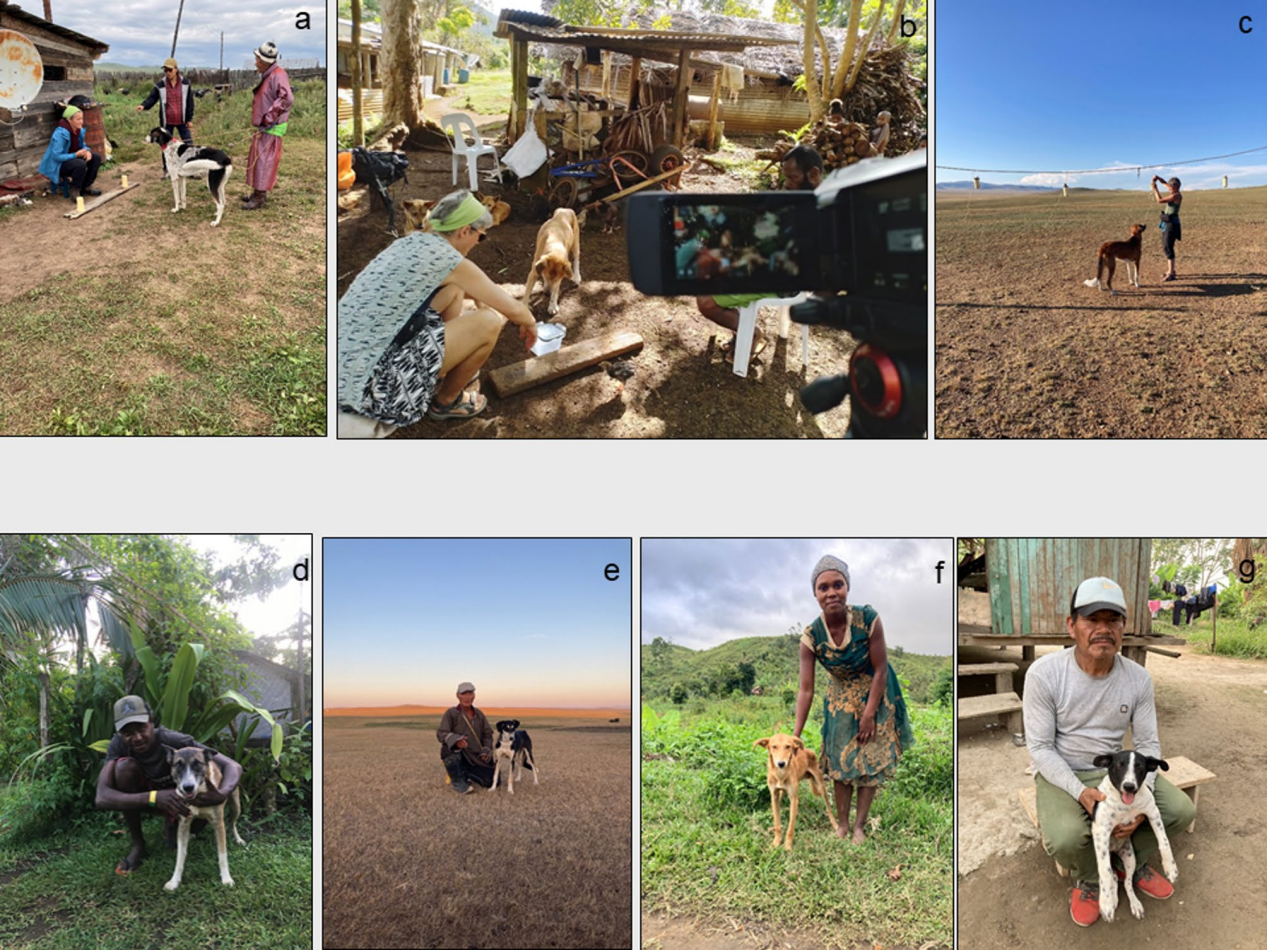
Testing situation and hunters with their dogs - examples from each of the four non WEIRD societies: **(a)** Pointing test in Mongolia, **(b)** Unsolvable Problem test in Vanuatu, **(c)** Showing test in Mongolia, **(d)** Vanuatu, **(e)** Mongolia, **(f)** Madagascar, **(g)** Peru.

**Fig. 2:**
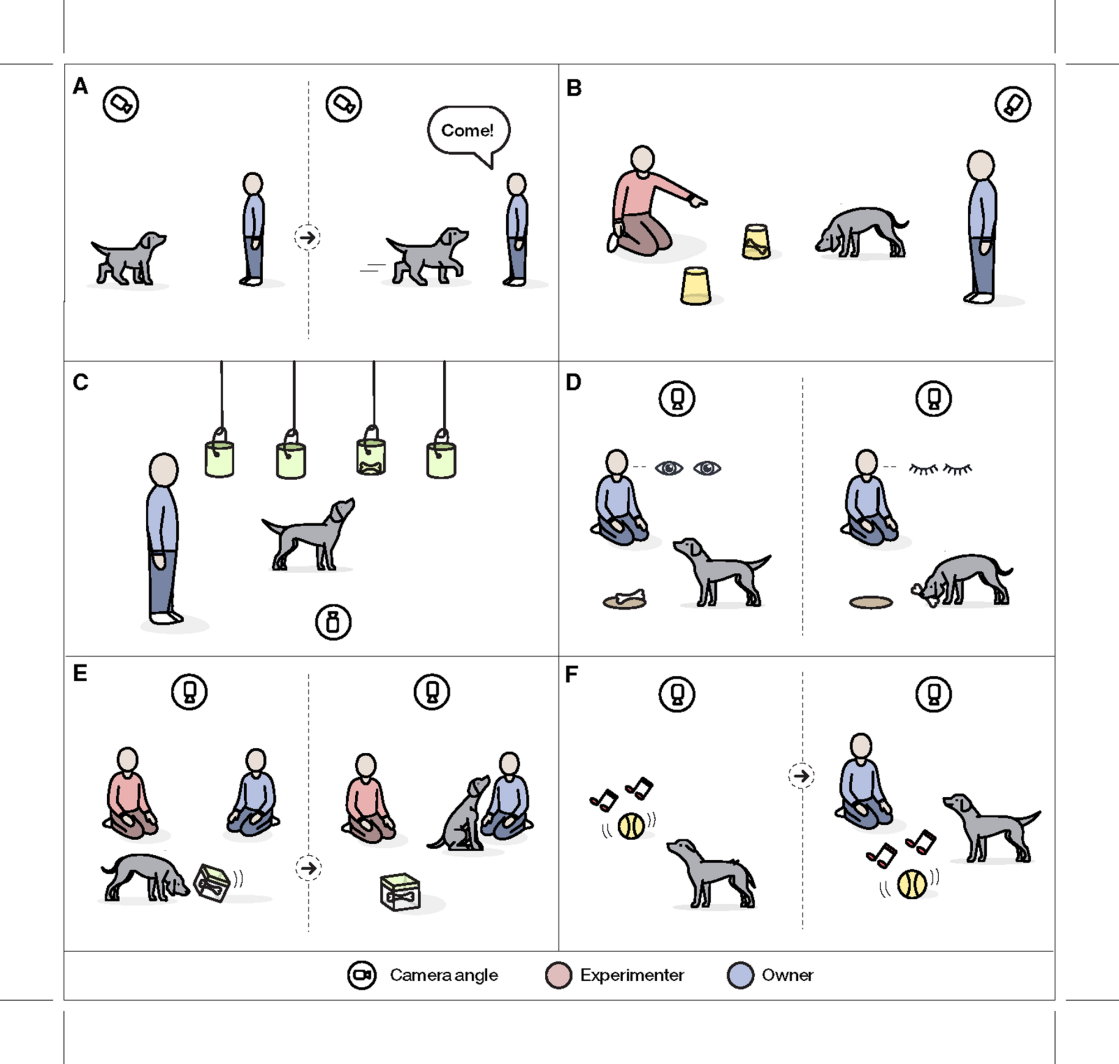
Setup of the six tests: **(a)** Obedience test, **(b)** Pointing test, **(c)** Showing test, **(d)** Perspective taking test, **(e)** Unsolvable problem test, **(f)** Social referencing test.

As function influences the dog–human relationship^27,28^, we focused on one function and tested hunting dogs (see Supplementary Materials for details about the subjects). We selected five rural societies in culturally diverse locations on different continents, four of which deliberately contrast with WEIRD criteria: Vanuatu, Mongolia, Madagascar, and Peru are non-WEIRD countries (Fig. 1), and Germany is a WEIRD country. These five study sites differed sharply in ecology, subsistence, hunting practice, and religion. In rural Germany, hunting dogs are very specialized, individually owned, and trained for obedience, as they have to pass hunting-related exams. In Vanuatu, dogs are central to hunting wild pigs in dense forest, where successful hunting depends heavily on owners’ ability to read their dogs’ signals. In Mongolia, dogs are kept in rural hunting and guarding contexts, as in other non-WEIRD settings, but they are generally less embedded in formal training regimes than German dogs. In Madagascar, dogs are crucial for the hunters in the dense forest, whereas in the Shipibo-Konibo communities in Amazonian Peru, hunters may sometimes hunt without them. In all five study sites, we presented at least 30 dog-owner dyads with six experiments from established paradigms that examined dog socio-cognitive abilities and dog–human communication (see Fig. 2). We tested obedience, use of the human pointing gesture, showing behavior, perspective taking, social referencing, and persistence and communication during an unsolvable problem. The questionnaire included questions about the bond to the dogs and how they are kept, including aspects that Chira et al. (2023) investigated^27^ (see Supplementary Materials).

## Results

### Questionnaire: How do owners describe the relationship to their dogs?

All owners in all five countries stated that they enjoy having their dog around at least some of the time and that their life is sometimes better because they have a dog (except for one owner in Peru). A vast majority of the owners also reported that they could at least sometimes rely on their dog to be there for them (> 90% in all countries; no significant variation across countries: *χ*^2^(4) = 3.32, *p* = 0.505) and that their dog would try to protect them at least sometimes in threatening situations (> 90% in all countries; no significant variation across countries: *χ*^2^(4) = 4.72, *p* = 0.317). There was significant variation in response to the question whether their dog would understand the owner’s emotions (sadness, fear) across countries (*χ*^2^(4) = 21.09, *p* < 0.001). Pairwise comparisons revealed that the owners in Peru were significantly less likely to say that their dogs understood their feelings than the ones in Germany (z = 3.12, *p* = 0.016) and Mongolia (z = 3.24, *p* = 0.011). The proportion of owners who reported at least sometimes regretting having a dog varied significantly across countries (*χ*^2^(4) = 40.55, *p* < 0.001). Owners in Peru were significantly more likely to answer that they at least sometimes regret to have a dog compared to the ones in the other countries (Germany – Peru: t = 3.50, *p* = 0.005; Madagascar – Peru: t = 3.22, *p* = 0.013; Mongolia – Peru: t = 3.77, *p* = 0.002; Vanuatu – Peru: t = 3.06, *p* = 0.022).

**Fig. 3:**
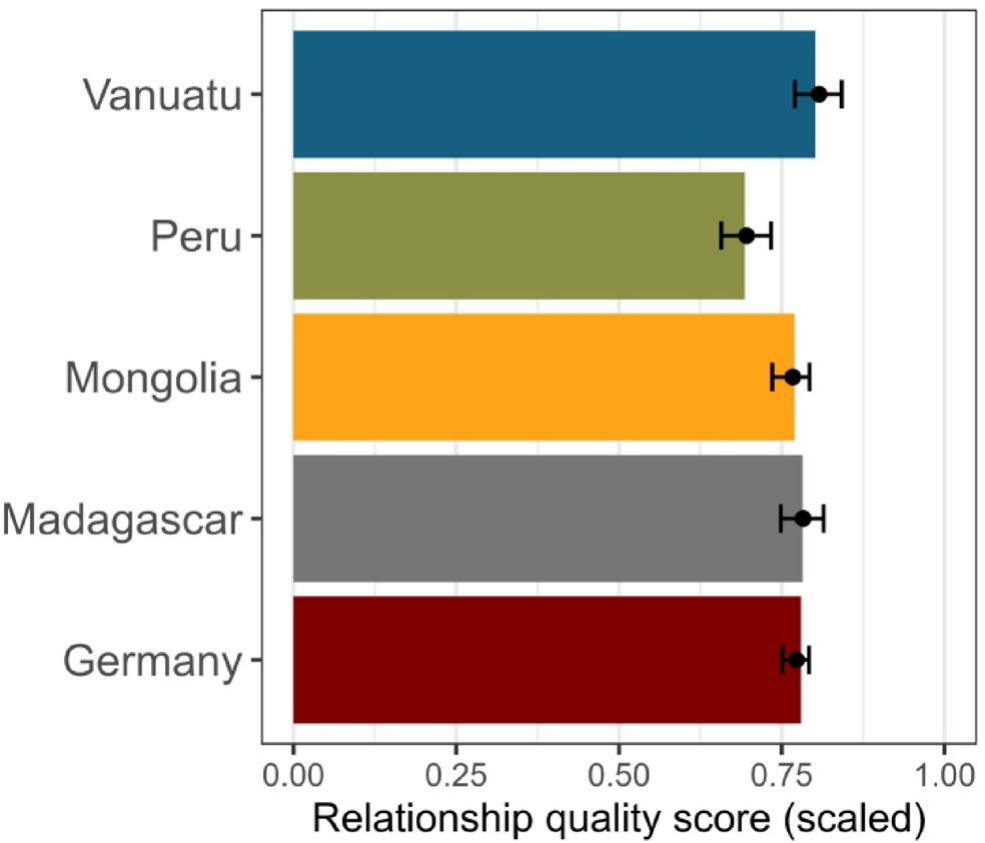
Visualization of the relationship score. The bars represent average relationship quality scores. The dots show the fitted values of a beta regression and the error bars represent the bootstrapped 95% CI.

The relationship quality score varied significantly across countries (*χ*^2^(4) = 22.97, *p* < 0.001). The owners in Peru rated their relationship quality worse than in all other countries (Germany – Peru: z = 3.27, *p* = 0.010; Madagascar – Peru: z = 3.72, *p* = 0.002; Mongolia – Peru: z = 3.00, *p* = 0.023; Vanuatu – Peru: z = 4.77, *p* < 0.001, see Fig. 3).

### Obedience test: Do dogs follow the command ”Come here?”

Dogs in general followed the command: “Come here”.

**Fig. 4:**
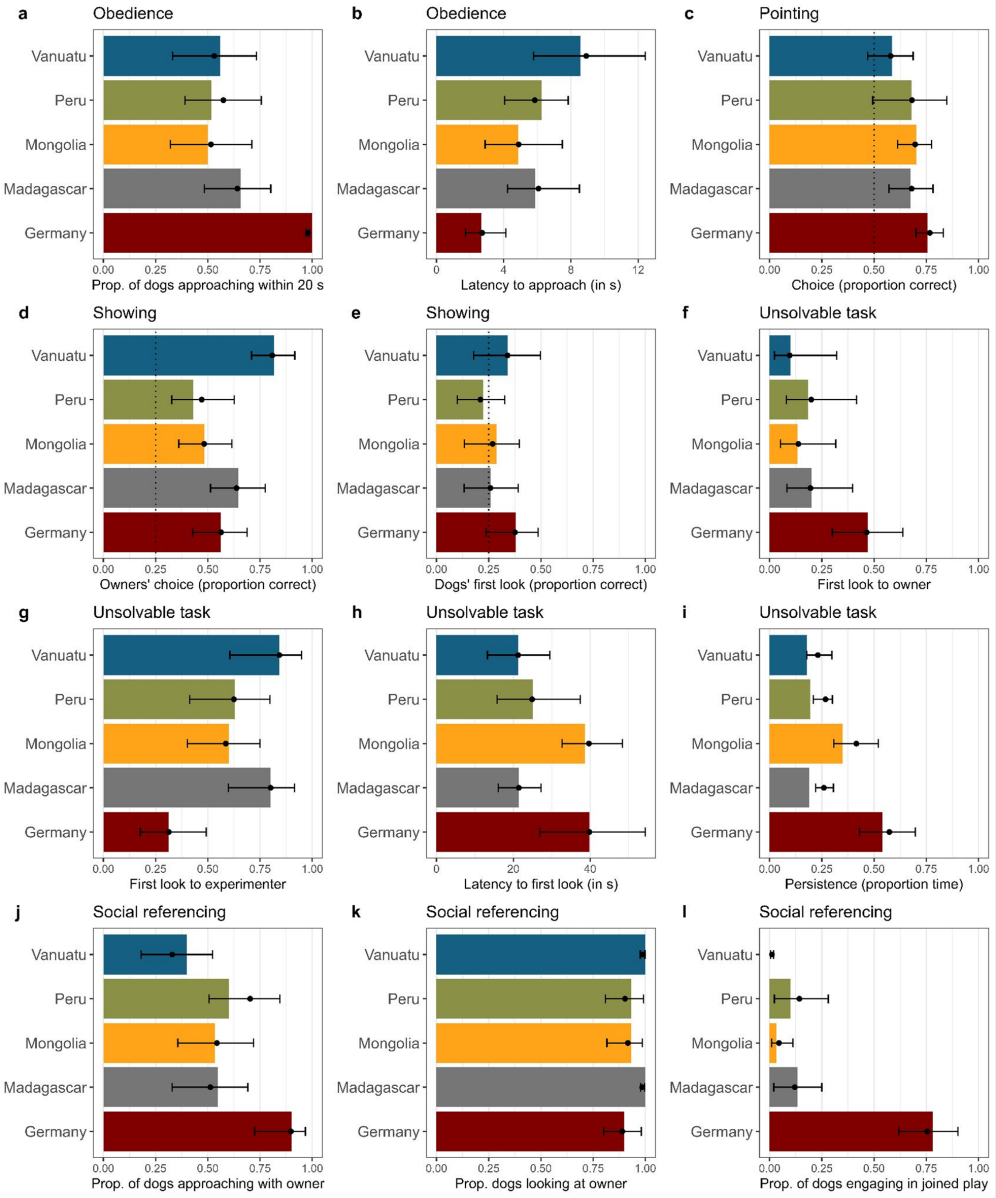
Performance in the 6 tests. **(a)** Obedience: Proportion of dogs that approached their owner within 20 s; **(b)** Obedience: Latency to approach owner; **(c)** Pointing: Choice of dogs; **(d)** Showing: Owners’ choice; **(e)** Showing: Dogs’ first look; **(f)** Unsolvable problem: Dogs’ likelihood to first look at owner; **(g)** Unsolvable problem: Dogs` likelihood to first look at experimenter; **(h)** Unsolvable problem: latency to first look at owner or experimenter; **(i)** Unsolvable problem: dogs’ persistence; **(j)** Social referencing: Dogs’ likelihood to approach with owner; **(k)** Social referencing: Dogs’ likelihood to look at owner; **(l)** Social referencing: Dogs’ likelihood to engage in joined play. The bars represent average performance levels, the dotted lines indicate chance level and the error bars represent the bootstrapped 95% CI. The dots show the fitted values of a Firth’s logistic regression (a, k,l), a gamma GLM (b, h), a binomial GLMM (c, d,e), a binomial GLM (f, g,j) and a beta GLM (i).

1. *Approaching Owner within 20s*: Although there was a significant variation across countries (χ2(4) = 20.48, *p* < 0.001, Fig. 4a), none of the pairwise comparisons between countries were significant after the correction for multiple comparisons. The relationship score did not significantly affect the dogs’ performance (χ^2^(1) = 1.91, *p* = 0.167).

2. *Approach latency*: We found significant variation across countries (χ2(4) = 17.49, *p* = 0.002, Fig. 4b). After controlling for multiple comparisons, German dogs approached significantly faster than the dogs in Madagascar (z = 3.02, *p* = 0.021), Vanuatu (z = 3.94, *p* < 0.001). Other comparisons were not significant. The relationship score did not significantly affect the dogs’ performance (*χ*^2^(1) = 0.51, *p* = 0.475).

### Pointing test: Do dogs follow the human pointing gesture to locate hidden food?

We replicated previous findings that dogs use the human pointing gesture.

*Dogs’ choice performance*: The populations’ performance did not vary significantly across countries (*χ*^2^(4) = 8.49, *p* = 0.075, Fig. 4c). The relationship score did not significantly affect the dogs’ performance either (*χ*^2^(1) = 0.29, *p* = 0.591). The dogs performed significantly above chance in all populations (Mean ± SEM: Germany: 75.6 ± 3.9%; Madagascar: 67.4 ± 5.1%; Mongolia: 70.2 ± 3.9%) except for Vanuatu (58.6 ± 6.7) and Peru (67.9 ± 6.0%). However, the small Peruvian sample of dogs with valid trials (*n* = 8) might explain their non-significant result.

### Showing test: Do dogs show owners the location of hidden food - and can owners interpret this showing behavior correctly?

We replicated previous findings that dogs and owners can communicate successfully about the location of hidden food.

1. *Owners’ choice performance*: The owners performed significantly above chance level of 25% in all five tested populations. There was significant variation across the five tested populations (*χ*^2^(4) = 17.56, *p* = 0.002, Fig. 4d) with the Vanuatu dog owners (mean ± se: 81.7 ± 4.5%) significantly outperforming the owners in all other countries (Germany: 56.3 ± 6.1%, z = 2.80, *p* = 0.041; Mongolia: 48.4 ± 6.0%, z = 3.54, *p* = 0.004; Peru: 43.1 ± 6.61%; z = 3.42, *p* = 0.006) except for the ones in Madagascar (64.5 ± 7.8%; z = 1.96, *p* = 0.284). No other comparisons were significant (all *p* > 0.1). The relationship score did not significantly affect the owners’ performance (*χ*^2^(1) = 0.11, *p* = 0.738).
2. *Dogs’ first look*: The dogs did not signal the correct container with their first look significantly above chance levels (Fig. 4e). Moreover, there was no significant variation in their first look across the five tested populations (*χ*^2^(4) = 4.39, *p* = 0.356). The relationship score did not significantly affect the dogs’ performance either (*χ*^2^(1) = 0.0004, *p* = 0.984).

### Perspective taking test: Do dogs avoid forbidden food when owners are watching them?

We did not replicate previous findings that dogs are differentiate between humans’ attentional state.

1. *Food taken by dog*: We found no significant interaction between country and condition (eyes open or closed; *χ*^2^(4) = 2.97, *p* = 0.563). We then fitted a reduced model to evaluate the main effects. Whether owners had their eyes open or closed did not significantly affect the dogs’ decision to take the food (*χ*^2^(1) = 0.08, *p* = 0.778). There was significant variation across the five tested populations in the dogs’ likelihood to steal (*χ*^2^(4) = 17.97, *p* = 0.001). However, when analyzing all pairwise comparisons, while controlling for multiple comparisons, none of the comparisons were significant. The relationship score did not significantly affect the dogs’ performance (*χ*^2^(1) = 0.45, *p* = 0.503).
2. *Dogs’ looks at owners while waiting*: We checked whether there was a significant interaction between country and condition but found none (*χ*^2^(4) = 7.42, *p* = 0.115). We then fitted a reduced model to evaluate the main effects. Whether owners had their eyes open or closed did not significantly affect the dogs’ likelihood to look at them (*χ*^2^(1) = 1.33, *p* = 0.248). There was significant variation across the five tested populations (*χ*^2^(4) = 22.55, *p* < 0.001), but none of the pairwise comparisons were significant after controlling for multiple comparisons (all *p* > 0.1). The relationship score did not significantly affect the dogs’ performance (*χ*^2^(1) = 1.91, *p* = 0.167).

### Unsolvable test: Do dogs look at humans when a problem becomes unsolvable?

We replicated previous findings that dogs look at humans when faced with an unsolvable problem.

1. *First look to owner*: We found significant variation across countries (χ2(4) = 12.88, *p* = 0.012, Fig. 4f) but no significant differences after controlling for multiple comparisons. The relationship score did not significantly affect the dogs’ looking behavior (χ2(1) = 0.22, *p* = 0.641).
2. *First look to experimenter*: We found significant variation across countries (χ2(4) = 20.74, *p* < 0.001, Fig. 4g). After controlling for multiple comparisons, German dogs looked significantly less to the experimenter first than the ones in Madagascar (z = 3.46, *p* = 0.005) and Vanuatu (z = 3.35, *p* = 0.007). The relationship score did not significantly affect the dogs’ looking behavior (χ2(1) = 0.01, *p* = 0.909).
3. *Latency to first look at experimenter or owner*: The first look latency varied significantly across countries (χ2(4) = 14.85, *p* = 0.005, Fig. 4h). The German dogs looked at the owner or experimenter significantly later than those from Madagascar (z = 2.81, *p* = 0.040). The relationship score did not significantly affect the dogs’ first look latency (χ2(1) = 0.01, *p* = 0.916).
4. *Persistence*: The proportion of time the dogs spent trying to open the box varied significantly across countries (χ2(4) = 35.55, *p* < 0.001, Fig. 4i). The German dogs were more persistent than the dogs from Madagascar (z = 5.25, *p* < 0.001), Peru (z = 4.98, *p* < 0.001), Vanuatu (z = 5.52, *p* < 0.001). The Mongolian dogs were also more persistent than those on Vanuatu (z = 2.94, *p* = 0.027). The relationship score did not significantly affect the dogs’ persistence (χ2(1) = 0.63, *p* = 0.429).

### Social referencing test: Do dogs use the information from a human about a novel potentially dangerous object?

We replicated previous findings that dogs are capable of social referencing.

1. *Approach with owner*: We found significant variation across countries (χ2(4) = 22.65, *p* < 0.001, Fig. 4j). After controlling for multiple comparisons, German dogs approached significantly more often with their owner than the ones in Madagascar (z = −2.93, *p* = 0.027), Mongolia (z = −2.75, *p* = 0.045), Vanuatu (z = −3.91, *p* < 0.001) but not Peru (z = −1.77, *p* = 0.384). The other comparisons were not significant. Additionally, a higher relationship score increased the likelihood that the dogs approached with their owners (χ2(1) = 10.70, *p* = 0.001).
2. *Looking at owner*: We found no significant variation across countries (χ2(4) = 4.78, *p* = 0.311, Fig. 4k). The relationship score did not significantly affect the dogs’ looking behavior (χ2(1) = 0.33, *p* = 0.565).
3. *Joined play*: We found significant variation across countries (χ2(4) = 67.10, *p* < 0.001, Fig. 4l). After controlling for multiple comparisons, German dogs play more often jointly with the owner than the ones in Madagascar (t = 4.58, *p* < 0.001), Mongolia (t = 4.47, *p* < 0.001), Peru (t = 4.13, *p* < 0.001) and Vanuatu (z = 3.76, *p* = 0.002). The relationship score did not significantly affect the dogs’ play behavior (χ2(1) = 2.85, *p* = 0.091).

## Discussion

Contrary to our first hypothesis that cultural differences exist in the dog–human relationship, we found many striking similarities across the five societies tested. In particular, owners universally valued their dogs, as they reported enjoying their dogs’ company, being able to rely on them, and that their dogs would protect them. Dogs from all societies also behaved similarly when using human distal pointing to locate hidden food^10^ and when showing food to the owner. Interestingly, we also found very similar-looking behaviour in all dog populations.

Dogs looked at humans in the Perspective Taking Test, in the Social Referencing Test and in the Unsolvable Problem Test. It is likely that dogs all over the world are constantly monitoring humans^36^. This enables them to gather information about potentially dangerous objects and assess whether their owner is attentive and open to communication. In the Perspective Taking Test, we were not able to replicate the findings that dogs avoid forbidden food when they are being watched^15^- probably due to problems with the setup in which the owner, rather than an experimenter, forbade dogs to eat the food in a less consistent way than in previous studies. However, the dogs’ looking behaviour towards their owners was similar in all five societies. There were also no significant differences between cultures in whether dogs looked at humans when faced with an unsolvable problem (although German dogs exhibited slightly different behaviour, as noted below). Whether dogs in this situation look at humans for help^18^ or whether increased looking behaviour is a consequence of reduced persistence^37,38^ is an ongoing debate.

Although we found many cross-cultural similarities in social-cognitive abilities, we also observed several differences between societies. In our view, differences in hunting techniques (see S6) and social conditions likely explain these. In the Showing Test, we found that - although dogs behaved similarly in all countries - owners in Vanuatu were better at interpreting their dogs’ behaviour than in the other countries. This can be explained by the fact that in Vanuatu, hunting dogs are crucial to finding prey (wild pigs) in the dense forest. We also found that the relationship score - which illustrates the closeness of the bond between dog and owner (see S4 for details) - was lower in Peru. This was the only society (except Germany) where hunters sometimes hunt without their dogs, as dogs are not essential to their hunting success.

In some respects, our results support Hypothesis 1b that the dog–human bond in Germany is different than in the non-WEIRD countries. Dogs were more obedient: they came to their owner faster in the Obedience test and tended to eat less forbidden food in the Perspective-taking test. When faced with an unsolvable problem, they were more persistent than dogs from other societies and looked mainly at the owner rather than at an unfamiliar person. This might suggest that dogs in WEIRD countries are more selected for persistence and that obedience training plays a greater role in their upbringing^20^. German hunting dogs may also be more accustomed to test situations, as they have to pass an exam. Our results align with the notion that Western dogs are more attached to a single owner, whereas dogs in non-WEIRD societies may not have such a preference. This fits well with the theory that WEIRD societies are more individualistic, whereas elsewhere, community plays a much greater role. According to Henrich et al. (2020), WEIRD people are psychologically different, i.e. more individualistic and control-oriented. Being more individualistic, WEIRD owners might prefer a one-to-one, personal and unique bond with their dog^39^. As a result, dogs in WEIRD societies are more likely to be attached to a single owner. This could have proximate reasons (i.e., individual dogs learn to be more attached to a single owner) but also ultimate reasons (i.e., there was a selection pressure for dogs in WEIRD societies toward the ability to form a close attachment to a single owner). Regarding non-WEIRD dogs, it is also likely that this environment favours more independent dogs. Although owners in all four non-WEIRD societies claimed in the interviews that they regularly feed their dogs, dogs often walk on their own, scavenging for food scraps (personal observation and communication) or even chase prey on their own^40,41^ without waiting for permission or direction.

In our second hypothesis, we predicted that the dog’s performance in the tests would depend on the closeness of the bond, as measured by a relationship score. This score was calculated based on the owners’ statements about their feelings towards their dogs and their activities with them, such as playing, walking, and grooming. Interestingly, this score (and also the answers to individual questions in the questionnaire) were, for the most part, not significant predictors of performance in the tests. One reason may be that the score was relatively high and did not vary much within or between societies (except in Peru, see Fig. 3). Thus, the bond between dogs and their owners was generally close. Only in the social referencing test did it have a significant effect: the higher the relationship score, the more likely the dogs were to approach the object with their owners in all five societies. This means that, when confronted with a fearful object, dogs are more likely to approach their owner if the bond is close and they spend a lot of time together.

One limitation of our study is that we focused on dogs with the function of hunting, which may be unique^27,28^. Thus, the question remains whether our findings are limited to hunting dogs. However, it should be noted that in the non-WEIRD societies, only a few of the dogs studied were used exclusively for hunting (8 out of 35 in Mongolia and 5 out of 32 in Peru). Almost all dogs in these countries were also used for guarding. Even in Germany, where dogs are more specialized^42^, 16 out of 34 hunting dogs had additional functions according to their owners. It is therefore likely that our results are not unique to hunting dogs. Furthermore, Germany was the only country representing WEIRD societies in our study. However, as these paradigms had previously been conducted in various WEIRD countries and the findings matched those of our current study with German dogs, we believe that our findings can be considered representative. Another limitation is that we were unable to test all dogs in all tests due to practical issues. In particular, in Peru, Vanuatu and Madagascar some dogs were not used to their owners holding them back or eat from cups and Tupperware, which were preconditions for the Pointing Test and the Unsolvable Problem Test. In Mongolia, Madagascar and Peru there were dropouts in the Perspective Taking Test, as dogs failed to follow the command to eat the forbidden food for some seconds – until the owner sat on the chair.

These problems also arose because we used tests developed for dogs in WEIRD countries in this initial investigation of cultural differences in dog–human interaction. In this way, we might not capture typical skills for non-WEIRD dogs. Similar problems arise for the questionnaire: (a) It could be that translations distort the question, and (b) there might be cultural differences in how owners answer questions and to what extent they give the answer the questioner wants to hear (social desirability bias).

Taken together, our results suggest more similarities than differences in dog–human interaction. Some authors have speculated that dogs and humans have evolved together as the social structure of the group and the hunting behaviour of their ancestors were similar^4,43,44^. Dogs were domesticated 30.000 years ago, much earlier than any other animal^45,46^. Domestication can be described as a mutualistic relationship, where both humans and domesticated animals benefit^47,48^. The advantage for the early dogs was access to a new food resource in human camps and protection from predators^4^. The benefit for humans was probably assistance during hunting^28,49,50^. As hunting requires cooperation, attentiveness, and trust among individuals, this may have favored the development of the close psychological bond that dogs are famous for in WEIRD countries^2,46^ see above) and that we have found in very distinct cultures all over the world. The special bond between dogs and humans may have contributed to the success of both species, enabling them to settle all around the globe^7,51^. The very substantial differences between cultures around the world do not appear to have a major influence on this bond, particularly when dogs are still used for hunting, which requires cooperation.

To further study these questions, experiments should be developed that better capture the everyday situations of non-WEIRD dogs, such as food competition, independent problem solving and alertness to environmental hazards. Other topics to investigate include dog obedience, which may vary depending on the living conditions in a particular culture. For example, we found that dogs in Madagascar were particularly obedient regarding forbidden foods whereas two thirds of the dogs in Peru did not obey the command not to eat the food. Dogs in non-WEIRD countries may exhibit better obedience in relevant tasks, such as a single call summoning the entire group of hunting dogs (common in Vanuatu).

## Materials and methods

### Overview study design

A test battery consisting of six behavioural tests was conducted in a cross-cultural sample to investigate various facets of dog cognition and the communication between dogs and their owners. The non-WEIRD societies were chosen to fit non-WEIRD criteria and capture the continents Asia, Oceania, Africa, South America and Europe. We tested dogs with their owners in rural Germany (*N* = 34), on the island of Efate in Vanuatu (*N* = 30), in the Khentii Province in Eastern Mongolia (*N* = 35), in the region Andasibe in Eastern Madagascar (*N* = 33) and at Shipibo-Konibo villages in the Amazon region of Peru (*N* = 32). Owners were recruited through local assistants. Dog-owner teams participated voluntarily in the study. All tested dogs were used for hunting, but most were also used for guarding, and sometimes other functions, see Table S1 for details. In most cases the tests were carried out in the owner’s backyards or gardens, but a few German dogs were tested in The DogStudies Lab.

Testing took place between October 2022 and September 2024. The whole project was ethically approved by the Ethical Counsel of the Max Planck Society (Application 2019_17). This approval included both, statements for human ethics and animal ethics. All methods are reported in accordance with ARRIVE guidelines. The animal research complies with the “Guidelines for the Treatment of Animals”. An informed consent was obtained from all participating dog owners for all conducted experiments and the questionnaires. The study was conducted in accordance with all relevant guidelines and regulations in Germany, Madagascar, Mongolia, Peru and Vanuatu. We also have obtained an informed consent to publish the images (Fig. 1) and Videos (Supplementary Materials) in an online open-access publication from all dog owners that could be identified. See Supplementary Materials for a detailed Ethics Statement.

### Design and procedure

We aimed to test all dogs in all six tests. However, in some cases dogs (or individual trials) had to be excluded for the following reasons: the dog was not accustomed to being held by the owner (Pointing and Unsolvable Problem), showed no hesitation in eating food when it was forbidden (Perspective Taking), did not eat from cups or Tupperware containers (Pointing and Unsolvable Problem), the owner refused to conduct the test (only in Germany), decreased food motivation, disappearance of the dog, or experimenter error. See Table S2 for the number of dogs that were successfully tested in each task in each country.

The six tests were presented in different orders depending on the practical issues such as shyness and food motivation of the subject, interference of other dogs and people and weather conditions. All tests were videotaped and filmed with a camera 4 to 5 m from the testing place to film both the owner and the dog. The camera followed the dog in case the dog left the filming area. The questionnaire was presented to the owner in two breaks between tests, so the dogs could relax. JB was experimenter (E) for all non-WEIRD dogs, and most of the German dogs. In all tests the owner (O) played a crucial role. The instructions were given before each test by local translators who were native speakers of the respective language. For details of the coded behavior, see Table S5.

*Questionnaire*: The questionnaire contained 31 questions regarding information about the dog, dog husbandry, behaviour, owner satisfaction, owner attachment towards the dog and the caregiving behavior of the dog. It is based on established questionnaires and the approach of Chira et al. (2023), see Tables S3 and S4. Six individual questions (S3) and a Relationship Quality Score (including 12 questions, see S4) were included in the analysis. The questionnaire was translated into the respective language. It was presented to the owners by local research assistants fluent in the given language. In most cases the local assistants read the questions to the dog owners, sometimes owners filled it out themselves with the assistant present to clear up any ambiguities. In addition, we interviewed one hunter in each society about the common hunting techniques (see Table S6 for details).

*Obedience test*: Here we tested whether dogs obeyed O`s “Come” and “Stay” command (for example “sit” or “Lay down”). O was asked to call the dog and to make it stay. Then O was asked to move 5 steps away while the dog was supposed to stay. After 20 s, O was asked to call the dog again. If the procedure did not work at the first trial, it could be repeated up to two more times. We measured whether O was approached within 20 s and the latency to approach. Dogs in the non-WEIRD societies did not know a command for “stay”; thus, we did not include that variable in the analysis.

*Pointing test* (based on^11^): Here we tested whether dogs use the human pointing gesture to locate hidden food. In this object choice task, E and O faced each other, with O holding the dog. In front of E, two cups were placed upside down on a wooden board or a concrete floor. During the pretest, the dog learned that food was under one of the two cups, and that after being released by O it had to select one cup and receive the food (for details, see Supplementary Materials). After the dog passed the pretest, the testing phase began. The two cups were placed in front of E but initially hidden behind a screen so the dog could not see where the food was hidden. The trial started when E showed the dog the food and hid it under one of the cups, following a predetermined randomized order. E then removed the screen and slid the cups apart from each other, positioning them to the left and right. To provide the communicative cue, E drew the dog’s attention by calling his/her name and then pointed to the cup containing the food, using an ipsilateral proximal dynamic pointing gesture. The dog was then released by O. The choice was revealed once it had selected a cup by touching it or getting within a range of 10 cm with its snout. The test phase consists of 6 such trials. If the dog chose the correct cup with the food, it was allowed to eat it. If it selected the wrong cup, E revealed the correct one, but the dog was prohibited from eating the food. In the few cases when dogs did not select a cup within 15 s, the trials were not included in the analysis.

*Showing test* (based on^14^): Here, we tested whether dogs could successfully communicate the location of hidden food to their owners. Four cups, which served as hiding places, were placed or hung in a row at a distance of approximately 1 m from each other at a height the dog could not reach. O was instructed to move from the testing site out of sight while E took the dog to the cups. E then showed the dog a big piece of food and put it in one of the four cups. E ensured that the dog was attentive and saw where the food was hidden. Once the food was hidden, E moved away from the testing site and called O. O now had to find the correct cup with the dog’s help. S/he was free to decide whether to talk to the dog, watch it, or proceed in any other way. The trial ended with O’s decision on a cup that s/he was allowed to open. If O selected the correct cup, they were allowed to give the food to the dog. If O could not grasp a potential cue of the dog, s/he was encouraged to guess where the food was hidden. The trial was repeated once, after a 5-minute break between trials, resulting in two trials. We coded whether O selected the correct cup and whether dogs first looked at the correct cup when the trial began.

*Perspective taking test (*based on^15^): Here we tested whether dogs could take the visual perspective of the owner. For that O received two food pieces from E. First O gave the dog one piece. Then O placed the second piece on the floor and commanded the dog not to eat this food. O then sat down on a chair, 1.5 m away from the food, while facing it. O then behaved depending on the condition: s/he either kept the eyes open or closed. Importantly, after sitting on the chair, O was not allowed to react to the behavior of the dog, for example, by repeating the command. The trial ended when either the dog ate the food or after 60 s, when the food was removed from the floor. Each dog was presented with two trials, presented in randomized order. Dogs were excluded from the experiment when they did not obey the command not to eat the food on the floor until O sat on the chair. We coded whether dogs ate the forbidden food and whether they looked at O during the trial.

*Unsolvable problem test (*based on^19^): Here, we tested how persistent dogs are and whether they look at humans when faced with an unsolvable problem. In front of E was a transparent plastic box (tupperware), which could be securely closed with a lid. E and O either both sat or stood opposite each other, with O holding the dog. E showed the dog a piece of food, which was then placed inside the box. In the first trials, the lid was only put on the box without attaching it. Then the dog was released and could remove it and access the food. If the dog did not know how to access the food, E and O encouraged it. The test trial began after the dog successfully accessed the food thrice in a row. The problem became unsolvable for the dog as E attached the lid to the plastic box. The dog was allowed for 120 s to interact with the closed box. O and experimenter remained seated throughout this period, observing the dog without engaging it. After 120 s, E opened the box, and the dog received the food. We coded how long dogs interacted with the plastic box (persistence), the latency until they first looked at a human and whether they looked to O or E.

*Social referencing test* (based on^16^): Here, we tested whether dogs seek information from owners when they are faced with a scary object. An unfamiliar, scary toy was placed before the dog while O was absent. E activated the toy that moved and produced a melody. The dog could then approach and explore or avoid it. After 20 s, O entered the testing area and was instructed to approach the toy and encourage the dog to follow and interact with it. We coded whether dogs looked at O, approached the object with O, and played with the object.

### Statistical analysis

For the binary choice performance (showing task, perspective taking, pointing), questionnaire answers and look variables (showing task, perspective-taking, unsolvable task), we fitted a binomial GLMM or GLM (library lme4) with the test predictor country and the z-transformed control predictor trial number (in tasks with more than one trial per participant). For the GLMMs, we added the random intercept of subject ID and if possible the random slopes of the predictor variables.

In six instances, we used Firth’s Logistic Regression instead of a binomial GLM due to complete separation issues (obedience task: successful coming within 20 s; social referencing: looking at owner; social referencing: joined play; questionnaire: owner relies on dog, dog provides protection, dog understands emotions).

For latency variables (obedience task – approach latency; unsolvable task – first look latency) we fitted gamma GLMs (library lme4) with log link. For proportions (perspective taking – proportion time; relationship quality, unsolvable task – persistence) we fitted a beta GLMM (library glmmTMB) or GLM (library betareg) with the test predictor country and the z-transformed control predictor trial number (in tasks with more than one trial per participant). Where applicable, we added the random intercept of subject ID and included condition and the interaction between condition and country in the model (perspective-taking).

Pairwise comparisons between countries were corrected for multiple comparisons using Tukey contrasts (library multcomp and emmeans). For all models, we checked collinearity which was not an issue (max VIF: <1.1 for all models).For the beta and gamma models we also checked for overdispersion which was no issue (dispersion parameter: perspective-taking – latency: 1.27; unsolvable task: first look latency: 0.63; unsolvable task: persistence: 0.93; obedience – successful approach latency: 0.79; relationship quality: 1.08).

## Supplementary Information

Below is the link to the electronic supplementary material.

Supplementary Material 1

Supplementary Material 2

Supplementary Material 3

Supplementary Material 4

Supplementary Material 5

Supplementary Material 6

Supplementary Material 7

Supplementary Material 8

## Data Availability

All data and code used in the analyses are available on a public repository (https://doi.org/10.5281/zenodo.15516435).
