# Supplementary material for: Striking global similarities in dog–human interactions
Source: Sci Rep. 2026 Jun 23;16:18527. doi: 10.1038/s41598-026-57657-1 (PMC13291251; doi:10.1038/s41598-026-57657-1)
Supplement: Supplementary file 8 — Supplementary Material 8 [file 41598_2026_57657_MOESM8_ESM.doc]

Supplementary Materials for

**Striking Global Similarities in Dog-Human Interactions**

Juliane Bräuer *et al.*

*Corresponding author. Email: juliane.braeuer@uni-jena.de

**This PDF file includes:**

Supplementary Text

References

Tables S1 to S44

**Supplementary Text**

**Sample**

An a priori power analysis was conducted using G*Power 3 ^1^ to determine the required sample size for detecting between-country differences. The power analysis was based on an F-test (ANOVA) as an approximation of the planned between-group comparisons. Assuming six groups of equal size, an alpha level of .05, and a power of .80, a sample size of 26 dogs per group was estimated to detect a medium-to-large effect size.

Prior to testing, the local assistants who recruited the owners went to the villages to advertise the test, or advised local contact persons to do so. During these meetings, appointments were arranged and telephone numbers exchanged so that the owners could be contacted later. We chose villages with the same cultural background and language. Most owners of hunting dogs in the villages we visited agreed to participate in the test. Therefore, we assume that our sample was representative of that culture.

All dogs were used for hunting and owned by one household. Nearly all dogs had been living with their owners since they were a puppy. We only tested dogs that appeared healthy. Thus, we rejected dogs with open wounds, eye problems or limp.

Table S1 summarizes details of the subjects, including gender, age and breed.

**Ethics Statement**

The project Cultural Differences in Dog-Human Interactions received approval from the Ethics Council of the Max Planck Society on 13 August 2019 (Application No: 2019_17). Participation was voluntary. Participants signed a consent form, which informed them about their involvement in the project and the processing of their data. The collected data were only utilized for scientific purposes. Participants could complete an additional consent form if they agreed to allow photo and video footage of themselves and their dogs to be used for illustrative purposes, such as presentations and publications. Owners were free to withdraw from the participation at any time. After testing each dyad we gave a compensation present to the owner, which was worth about 8-10 EUR. These presents were chosen depending on what local helpers had advised us to be appreciated by the dog owners (Vanuatu: machetes, Mongolia: head lamps and knifes, Madagascar: rice bags and oil, Peru: bag with long-life food, Germany: dog toy or dog food).

**Materials**

The test battery was purposefully created to be feasible in remote places, using materials easy to transport. All trials were documented on film with one camera (Panasonic HC-V785) on a portable tripod.

Opaque coffee mugs with a lid served as hiding places in both the Showing and the Pointing test. The Pointing test also required the usage of a barrier to block the dog's sight when hiding the food, as well as a wooden board as a base to slide the cups around and chairs for the owner and the experimenter. For the Unsolvable Problem test a transparent food storage container (20x15x9cm) with holes in the lid was used, so the dogs were able to smell the food. For the Social Referencing test, we used an animated dog toy (diameter 13cm) that bounced back and forth and produced a noise. Dogs were rewarded with food, depending on what was common in a given society and on dog owners preferences (Vanuatu: fresh meat, tinned tuna; Mongolia: fresh meat, dog food; Madagascar: dog food, rice; Peru: dog food, dry fish; Germany: dog food). Optionally during some tests, owners were provided with a collar and leash in case they wanted to restrain their dogs. However, dogs were often less stressed when the owner held them close with their hands around their shoulders.

**Details of Procedure in the Pointing test**

In order to participate in the Pointing Test, dogs first had to pass a pretest. In the pretest, experimenter and owner faced each other, with the owner holding the dog either by a leash, a collar or by the shoulders. The two opaque cups were placed upside down on a wooden board by the experimenter. She gained the dog's attention and showed it a piece of food, which was then placed under one of the cups so the dog would see where it was hidden. The cups were then pushed apart, one to the left and on to the right. After a command given by the experimenter, the owner released the dog, so that it could select the correct cup with the food by touching it or getting within a 10 cm range of it with its snout. If the dog failed to do so, the experimenter showed the dog the food again. The procedure was repeated using the same cup. To pass the pretest, the dog had to successfully complete four trials in a row without errors, with the food being placed twice under the right cup and twice under the left cup.

**Questionnaire**

The questionnaire given to the owners included 31 items. It is based on established questionnaires by ^2-5^ and the approach of Chira et al. (2023) ^6^.

Tables S3 and S4 give an overview of the items used for our analysis.

**References**

| **Dog ID** | **Group** | **sex** | **age** | **breed** | **Additional function** |
| --- | --- | --- | --- | --- | --- |

| but_p_german | Germany | M | 5 | Small Munsterlander | guarding |
| --- | --- | --- | --- | --- | --- |
| iwa_k_german | Germany | M | 9 | Wirehaired Fox Terrier | none |
| lie_k_german | Germany | F | 1 | Wirehaired Fox Terrier | none |
| glo_v_german | Germany | F | 2 | German Wirehaired Pointer | guarding |
| ger_german | Germany | M | 4 | English Springer Spaniel | none |
| kir_b_german | Germany | F | 1 | Small Munsterlander | guarding, family dog |
| uma_p_german | Germany | F | 7 | Labrador | none |
| lai_m_german | Germany | F | 2 | Magyar Vizsla | family dog |
| wen_t_german | Germany | F | 5 | Irish Red Setter | none |
| que_g_german | Germany | F | 4 | Small Munsterlander | guarding, visiting dog |
| gin_j_german | Germany | F | 2 | German Longhaired Pointer | guarding |
| fae_j_german | Germany | F | 3 | German Longhaired Pointer | none |
| abr_c_german | Germany | M | 3 | German Longhaired Pointer | entertainment |
| gin_r_german | Germany | M | 1 | German Longhaired Pointer | none |
| blu_j_german | Germany | F | 9 | German Longhaired Pointer | none |
| fes_g_german | Germany | M | 4 | German Wirehaired Pointer | guarding, pulls the sled |
| oly_g_german | Germany | F | 7 | Slovensky Kopov | none |
| zor_s_german | Germany | F | 1 | Bavarian Mountain Scent Hound | guarding |
| afr_e_german | Germany | F | 3 | German Wirehaired Pointer | guarding |
| fan_german | Germany | M | 2 | Hanoverian Welding Dog | none |
| rub_s_german | Germany | M | 2 | German Quail Dog | none |
| wil_k_german | Germany | M | 2 | German Wirehaired Pointer | none |
| irm_m_german | Germany | F | 4 | Slovensky Kopov | guarding |
| wil_n_german | Germany | M | 1 | Border Terrier | entertainment, family dog |
| bas_n_german | Germany | M | 2 | German Shorthaired Pointer | entertainment, family dog |
| jef_german | Germany | M | 3 | Bavarian Mountain Scent Hound | guarding |
| bru_j_german | Germany | M | 4 | Labrador | guarding, entertainment, waste disposal |
| mir_l_german | Germany | M | 12 | Wirehaired Dachshund | guarding |
| jag_g_german | Germany | M | 8 | German Quail Dog | guarding, family dog, pulls bicycle |
| rub_k_german | Germany | F | 2 | German Quail Dog | none |
| ell_b_german | Germany | F | 2 | Small Munsterlander | guarding |
| isg_s_german | Germany | F | 3 | German Longhaired Pointer | none |
| amy_e_german | Germany | F | 7 | Parson Russel Terrier | none |
| car_e_german | Germany | M | 1 | Parson Russel Terrier | none |
| bal_r_vanuatu | Vanuatu | M | 7 | na | guarding, herding, companion |
| bea_p_vanuatu | Vanuatu | M | na | na | guarding |
| ben_n_vanuatu | Vanuatu | M | 1 | na | guarding, herding |
| bel_s_vanuatu | Vanuatu | F | 1 | na | guarding |
| bla_k_vanuatu | Vanuatu | F | 1 | na | guarding |
| bla_b_vanuatu | Vanuatu | M | na | na | guarding |
| bul_r_vanuatu | Vanuatu | M | 1 | na | guarding |
| cap_b_vanuatu | Vanuatu | M | na | na | guarding |
| ere_j_vanuatu | Vanuatu | F | na | na | guarding |
| hun_t_vanuatu | Vanuatu | M | na | na | guarding, follows owner to garden |
| jan_t_vanuatu | Vanuatu | F | 7 | na | guarding |
| pik_s_vanuatu | Vanuatu | M | na | na | guarding, protects game meat from other dogs |
| pol_e_vanuatu | Vanuatu | M | 4 | na | guarding |
| pre_j_vanuatu | Vanuatu | F | 3 | na | guarding |
| pri_c_vanuatu | Vanuatu | M | 1 | na | guarding, follows owner to garden |
| pup_s_vanuatu | Vanuatu | M | 3 | na | guarding |
| rab_j_vanuatu | Vanuatu | M | 6 | na | guarding, follows owner to beach to play |
| ram_r_vanuatu | Vanuatu | F | 2 | na | guarding, follows owner to garden |
| ron_j_vanuatu | Vanuatu | M | 3 | na | guarding, follows owner to garden |
| sni_d_vanuatu | Vanuatu | M | 2 | na | guarding |
| sno_l_vanuatu | Vanuatu | M | 1 | na | guarding, catches fowls in the yard when owner gives command (dog does not kill the fowls) |
| sto_t_vanuatu | Vanuatu | M | 1 | na | guarding |
| tal_j_vanuatu | Vanuatu | F | 4 | na | guarding |
| tig_b_vanuatu | Vanuatu | M | 8 | na | guarding, follows owner to garden |
| twi_k_vanuatu | Vanuatu | F | 3 | na | guarding, follows owner to garden |
| twi_kv_vanuatu | Vanuatu | F | na | na | guarding |
| whi_k_vanuatu | Vanuatu | F | na | na | guarding |
| zuk_o_vanuatu | Vanuatu | M | na | na | guarding, follows owner to garden |
| nav_k_vanuatu | Vanuatu | M | na | na | guarding |
| may_a_vanuatu | Vanuatu | F | 11 | na | guarding |
| ala_ba_mongolia | Mongolia | M | 5 | Mongolian Taiga | none |
| ala_bu_mongolia | Mongolia | M | 2 | Mongolian Taiga | guarding, herding |
| ala_l_mongolia | Mongolia | M | 1 | Mongolian Taiga | guarding, herding |
| ars_e_mongolia | Mongolia | M | 4 | Mongolian Taiga | guarding |
| bar_e_mongolia | Mongolia | M | 7 | Mongolian Taiga | guarding |
| bur_s_mongolia | Mongolia | F | 5 | Mongolian Taiga | guarding, herding |
| bur_b_mongolia | Mongolia | M | 10 | Mongolian Taiga | guarding |
| bur_o_mongolia | Mongolia | M | 2 | Mongolian Taiga | none |
| buu_g_mongolia | Mongolia | M | 1 | Mongolian Taiga | none |
| dii_l_mongolia | Mongolia | F | 3 | Mongolian Taiga | guarding, herding |
| erl_p_mongolia | Mongolia | M | 2 | Mongolian Taiga | herding, status symbol |
| jig_t_mongolia | Mongolia | M | 2 | Mongolian Taiga | guarding, herding |
| kha_n_mongolia | Mongolia | F | 1 | Mongolian Taiga | none |
| kha_e_mongolia | Mongolia | M | 2 | Mongolian Taiga | guarding, herding |
| kha_l_mongolia | Mongolia | M | 1 | Mongolian Taiga | guarding, status symbol |
| khu_e_mongolia | Mongolia | M | 6 | Mongolian Taiga | guarding, herding |
| khu_ge_mongolia | Mongolia | M | 4 | Mongolian Taiga | guarding |
| khu_gz_mongolia | Mongolia | F | 1 | Mongolian Taiga | guarding, herding |
| khu_d_mongolia | Mongolia | M | 2 | Mongolian Taiga | none |
| mon_m_mongolia | Mongolia | M | 4 | Mongolian Taiga | guarding |
| nac_t_mongolia | Mongolia | M | 3 | Mongolian Taiga | guarding, herding |
| sha_t_mongolia | Mongolia | F | 1 | Mongolian Taiga | guarding |
| sha_u_mongolia | Mongolia | F | 5 | Mongolian Taiga | guarding, herding |
| sha_j_mongolia | Mongolia | F | 1 | Mongolian Taiga | guarding, herding, status symbol |
| sho_s_mongolia | Mongolia | M | 2 | Mongolian Taiga | none |
| sum_g_mongolia | Mongolia | F | 5 | Mongolian Taiga | guarding, status symbol, entertainment |
| sun_p_mongolia | Mongolia | na | 1 | na | na |
| tsa_t_mongolia | Mongolia | M | 1 | Mongolian Taiga | guarding, entertainment |
| tsa_p_mongolia | Mongolia | M | 2 | Mongolian Taiga | guarding, herding |
| tse_s_mongolia | Mongolia | F | 4 | Mongolian Taiga | guarding |
| zev_a_mongolia | Mongolia | F | 1 | Mongolian Taiga | none |
| gun_g_mongolia | Mongolia | F | 4 | Mongolian Taiga | guarding, herding |
| kha_b_mongolia | Mongolia | M | 2 | Mongolian Taiga | guarding, herding |
| kha_m_mongolia | Mongolia | M | 2 | Mongolian Taiga | none |
| kha_ba_mongolia | Mongolia | M | 3 | Mongolian Taiga | guarding |
| bal_m_madagascar | Madagascar | F | 0.5 | Mix | guarding |
| big_s_madagascar | Madagascar | F | 2 | Border Collie Style | guarding |
| bla_d_madagascar | Madagascar | M | 4 | Greyhound | guarding |
| bor_a_madagascar | Madagascar | M | 3 | Mix | guarding |
| bru_p_madagascar | Madagascar | M | 10 | Mix | guarding |
| cap_j_madagascar | Madagascar | F | 14 | Mix | guarding |
| kay_madagascar | Madagascar | M | 5 | Mix | guarding |
| fot_t_madagascar | Madagascar | M | 7 | Mix | guarding |
| mbi_j_madagascar | Madagascar | F | 4 | Mix | guarding |
| mel_f_madagascar | Madagascar | M | 3 | Greyhound-Mix | guarding |
| mer_r_madagascar | Madagascar | F | 2 | Mix | guarding |
| mil_f_madagascar | Madagascar | F | 3 | Mix | guarding |
| mir_d_madagascar | Madagascar | M | 0.5 | Mix | guarding |
| mis_t_madagascar | Madagascar | M | 6 | Mix | guarding |
| pit_f_madagascar | Madagascar | M | 0.5 | Mix | guarding |
| qui_s_madagascar | Madagascar | M | 4 | Mix | guarding |
| rai_a_madagascar | Madagascar | M | 4 | Mix | guarding |
| rex_t_madagascar | Madagascar | M | 8 | Mix | guarding |
| rox_s_madagascar | Madagascar | M | 1 | Mix | guarding |
| tot_e_madagascar | Madagascar | M | 3 | Greyhound-Mix | guarding |
| tot_to_madagascar | Madagascar | F | 5 | Mix | guarding, help for agriculture |
| tot_d_madagascar | Madagascar | F | 5 | Mix | guarding |
| bil_a_madagascar | Madagascar | F | 2 | Mix | guarding |
| bla_n_madagascar | Madagascar | F | 10 | Mix | guarding |
| kap_j_madagascar | Madagascar | F | 4 | Yellow Mix | guarding |
| mil_p_madagascar | Madagascar | M | 4 | Mix | guarding, alarm to wake the owner |
| tot_th_madagascar | Madagascar | F | 2 | White Mix | guarding |
| tot_j_madagascar | Madagascar | M | 3 | Mix | guarding |
| tot_r_madagascar | Madagascar | M | 0.5 | Mix | guarding |
| tot_o_madagascar | Madagascar | M | 5 | Mix | guarding |
| tsi_o_madagascar | Madagascar | F | 3 | Mix | guarding |
| tsa_a_madagascar | Madagascar | M | 6 | Big Yellow Mix | guarding |
| rex_s_madagascar | Madagascar | M | 2 | Mix | guarding |
| aby_g_peru | Peru | F | 2 | Mix | guarding |
| alg_m_peru | Peru | M | 2 | Mix | carries loads |
| bau_l_peru | Peru | M | 2 | Mix | carries loads, waste disposal |
| bet_peru | Peru | M | 3 | Mix | none |
| big_g_peru | Peru | M | 2 | Mix | entertainment |
| bla_r_peru | Peru | M | 3 | Mix | none |
| cha_s_peru | Peru | M | 2 | Mix | carries loads, waste disposal |
| chi_c_peru | Peru | F | 0.5 | Mix | entertainment |
| coc_peru | Peru | F | 1 | Mix | none |
| dim_s_peru | Peru | M | 4 | Mix | none |
| dok_c_peru | Peru | M | 1 | Mix | none |
| her_l_peru | Peru | M | 1 | Mix | entertainment, rescue |
| loc_c_peru | Peru | F | 2 | Mix | carries loads, entertainment, waste disposal |
| loc_m_peru | Peru | F | 3 | Mix | none |
| lok_s_peru | Peru | M | 2 | Mix | none |
| lol_m_peru | Peru | F | 0.5 | Mix | none |
| lor_s_peru | Peru | M | 1 | Mix | entertainment |
| may_m_peru | Peru | F | 3 | Mix | waste disposal |
| neg_u_peru | Peru | na | na | na | na |
| neg_d_peru | Peru | F | 2 | Mix | entertainment |
| neg_v_peru | Peru | M | 3 | Mix | none |
| oso_r_peru | Peru | M | 1 | Mix | none |
| pal_f_peru | Peru | F | 1 | Mix | carries loads, entertainment, rescue |
| par_f_peru | Peru | F | 3 | Mix | none |
| pel_i_peru | Peru | F | 0.5 | Mix | none |
| rab_l_peru | Peru | F | 0.5 | Mix | entertainment |
| sen_g_peru | Peru | M | 2 | Mix | waste disposal |
| sik_f_peru | Peru | M | 4 | Mix | none |
| son_peru | Peru | M | 9 | Mix | none |
| tes_n_peru | Peru | M | 1 | Mix | none |
| tob_c_peru | Peru | M | 7 | Mix | entertainment |
| zoe_r_peru | Peru | F | 2 | Mix | entertainment |

**Table S1. Sample details.**

List of group/culture, sex, breed, age and additional functions of the hunting dogs in the sample. M = male, F = female. Age in years.

|  | Obedience | Pointing | Showing | Perspective Taking | Unsolvable Problem | Social referencing |
| --- | --- | --- | --- | --- | --- | --- |
| Germany | 34 | 34 | 34 | 34 | 33 | 33 |
| Vanuatu | 28 | 25 | 30 | 30 | 20 | 30 |
| Mongolia | 33 | 31 | 31 | 31 | 30 | 33 |
| Madagascar | 33 | 20 | 30 | 27 | 26 | 31 |
| Peru | 31 | 11 | 29 | 9 | 29 | 30 |

**Table S2. Sample sizes by test and country.**

Sample sizes for each test by country, only including dogs that participated successfully and without any mistakes in the test.

| Variable | Item | Response Codes |
| --- | --- | --- |
| owner_likes | I like it when my dog is around. | 0 = no; 1 = yes |
| life_better | My life is better because I have a dog. | 0 = no; 1 = yes |
| owner_relies | I can rely on my dog to be there for me. | 0 = no; 1 = yes |
| owner_protection | My dog tries to protect me in threatening situations. | 0 = no; 1 = yes |
| owner_feelings | My dog understands my emotions (sadness, fear). | 0 = no; 1 = yes |
| owner_regrets | Sometimes I wish that I would not have a dog. | 0 = no; 1 = yes |

**Table S3. Overview of items in the questionnaire included for individual comparisons.**

Items are in the order of appearance in the results. In the questionnaires we distinguished between 0 = no; 1 = sometimes; 2 = regularly, for the analysis we combined 1 and 2 to yes.

| Variable | Item | Scoring |
| --- | --- | --- |
| Function_relationship | Owner stated additional function of the dog beyond hunting on questionnaire | 0 = no; 1 = yes |
| walking | Do you walk your dog? | 0 = no; 1 = sometimes or regularly |
| playing | How often do you play with your dog? | 0 = no; 1 = sometimes or regularly |
| grooming | How often do you groom/wash your dog? | 0 = no; 1 = sometimes or regularly |
| death | When my dog dies it will be: like losing a person; sad; or annoying | 0 = sad or annoying; 1 = like losing a person |
| owner_relies | I can rely on my dog to be there for me. | 0 = no; 1 = sometimes; 2 = yes |
| owner_likes | I like it if my dog is around. | 0 = no; 1 = sometimes; 2 = yes |
| owner_feelings | My dog understands my emotions (sadness, fear). | 0 = no; 1 = sometimes; 2 = yes |
| owner_protection | My dog tries to protect me in threatening situations | 0 = no; 1 = sometimes; 2 = yes |
| dog_follows | My dog would always follow me around if he could. | 0 = no; 1 = sometimes; 2 = yes |
| life_better | My life is better because I have a dog. | 0 = no; 1 = sometimes; 2 = yes |
| owner_regrets | Sometimes I wish that I would not have a dog. | 2 = no; 1 = sometimes; 0 = yes |

**Table S4. Overview of items composing the Relationship Quality Score**.

The Relationship Quality Score is a sum score based on the additive coding of the responses listed in the "Scoring" column.

**Details of Coding**

|  | Behavior | Definition | Inter-observer-reliability r_s_ |
| --- | --- | --- | --- |
| Obedience | Approaching Owner within 20s | Occurrence: Whether dog came close to the owner (within range of 2 m) least once after owner called within 20 seconds. | κ = .88***, N = 33 |
|  | Approach Latency | Latency: time elapsed from the first “come" command (in the video) until the dog approached the owner in seconds. | ICC= .99***, N = 30 |
| Pointing Gesture | Dogs’ choice performance | Occurrence: whether the dog selected the correct cup that was baited with food and indicated by the experimenter. Selecting was defined by touching the cup or getting in a range within 10 cm with its snout. | κ = .91***, N = 177 |
| Showing Behavior | Owners’ choice performance | Occurrence: whether the owner chooses the correct cup. | Κ = 1.00***, N = 38 |
|  | Dogs’ first look | Occurrence: whether dog’s first look at a cup after owner appeared was directed at the correct cup. | κ = .74**, N = 38 |
| Perspective Taking | Food taken by Dog | Occurrence: whether the dog took the food. | κ = .85***, N = 55 |
|  | Dogs’ looks at owner while waiting | Occurrence: whether the dog looked at the owner at least once during the trial. | κ = .71***, N = 54 |
| Unsolvable Task | First look to owner | Occurrence: whether the dog looked away from the box for longer than one second and first directed its gaze at the owner. | κ = .84***, N = 36 |
|  | First look to experimenter | Occurrence: whether the dog looked away from the box for longer than one second and first directed its gaze at the experimenter. | κ = .84***, N = 36 |
|  | Persistence | Latency (in seconds) that the dog attempted to open the box, using either its mouth or paw, within a 15 cm range from the box. | ICC= .89***, N = 36 |
| Social Referencing | Looking at owner | Occurrence: whether the dog looked at the face of the owner. | κ = .94, N = 34 |
|  | Approach with owner | Occurrence: whether the dog got close to the scary object in the presence of the owner, sniffed at or touched it. | κ = .78, N = 34 |
|  | Joined play | Occurrence: whether the dog played with the scary object, including touching it and grabbing it in its mouth. | κ = .93**, N = 34 |

**Table S5. Coded behaviors for each test.**

Overview of variables, their definitions, and inter-observer-reliability scores for the behavioral tests. Inter-observer-reliability was calculated using Cohen's κ for categorical variables and intra-class correlation coefficients (ICC) for continuous variables. * p ≤ .05; ** p ≤ .01; *** p ≤ .001.

| Question/ Information | Vanuatu | | Mongolia | Madagascar | Peru | Germany |
| --- | --- | --- | --- | --- | --- | --- |
| **Frequency of hunting** | | 1x per week | Hunting season from October to December: several hunts for 3-5 days | 2x per week | 1x-2x per week, often for 3-4 days | 2x per week during hunting season |
| **Main Prey** | | Wild pig | Wild boar, antelope, deer, wolf | Tenrec | Turtle, monkey, caiman, capybara and more. | Mainly deer and wild boar |
| **Weapon** | | Machete (rarely gun or trap) | Gun, knife | Knife | Gun | Gun |
| **Number of dogs** | | At least 3 | Many, more than 1 | 1 | 1-2 | Depending on kind of hunt: 1-2 or 10-20 |
| **Number of hunters** | | 1 or many | Many, more than 1 | 1 | 1-2 | Depending on kind of hunt: 1-2 or 50 |
| **Special traditions** | | Dogs are borrowed to other hunters. | Traditional way to bury hunting dogs. | Dogs are fed before hunt. | Similar plant used for dogs and hunters for a successful hunt. | Exam is precondition for hunting dogs. |
| **Are dogs crucial for the hunt?** | | Yes. | Yes. | Yes. | No. | No. |
| **Hunting procedure** | | Dogs are used to follow the pig tracks. Dogs chase and surround pig, hunter kills pig. | First dogs on the leash follow the trail; hunter is on a horse. Then dogs are detached and start barking at game and sometimes kill game; otherwise hunter kills game. | Dogs bark, when there is a tenrec. Dogs dig tenrec out; they kill and fetch them. Hunters help. | Dogs follow track and find and chase the game; they bark to show the game. Hunters kill the game. | Hide hunts: dogs are used for tracking the game / Driven hunt: with drivers on the move, dog rummages up game alone, dogs should not kill the game. |
| **Extra information** | |  | "Dog and human share the same goal: to protect the herd and the home." | „Dogs like to help“ | „Dogs help because they like hunting.“ | “Dogs should understand that you hunt together, they should learn that it is a good shared experience. |

**Table S6. Hunter interviews.**

Overview of information from interviews with the hunters from different countries.

**Additional Results : Questionnaire**

| Country | Owner  relies | Owner  likes | Owner  feelings | Owner  protection | Life  better | Owner  regrets | Relationship  Quality Score  (Proportion) |
| --- | --- | --- | --- | --- | --- | --- | --- |
| Germany | 0.97 (0.03) | 1 (0) | 0.91 (0.05) | 0.94 (0.04) | 1 (0) | 0.15 (0.06) | 0.78 (0.01) |
| Madagascar | 0.94 (0.04) | 1 (0) | 0.82 (0.07) | 0.97 (0.03) | 1 (0) | 0.18 (0.07) | 0.78 (0.02) |
| Mongolia | 1 (0) | 1 (0) | 0.94 (0.04) | 1 (0) | 1 (0) | 0.03 (0.03) | 0.77 (0.02) |
| Peru | 0.9 (0.05) | 1 (0) | 0.53 (0.09) | 1 (0) | 0.97 (0.03) | 0.60 (0.09) | 0.69 (0.02) |
| Vanuatu | 0.97 (0.03) | 1 (0) | 0.70 (0.09) | 0.90 (0.06) | 1 (0) | 0 (0) | 0.80 (0.02) |

**Table S7.** **Descriptive statistics for questionnaire measures by country.**

Values are presented as means and standard errors (*M*(*SE*)). For wording of the questions and the rating scale, please see Table S3.

| Variable | χ² (4) | *p*-Value |
| --- | --- | --- |
| Owner relies | 3.32 | .505 |
| Owner Feelings | 21.09 | < .001*** |
| Owner Protection | 4.72 | .317 |
| Owner Regrets | 40.55 | < .001*** |
| Relationship Quality Score | 22.97 | < .001*** |

**Table S8. Likelihood ratio tests for country differences in questionnaire variables.**

χ² = likelihood ratio test statistic; df = 4. * *p* ≤ .05, ** *p* ≤ .01, *** *p* ≤ .001. Analyses of the variables “Owner likes” and “Life better” were not conducted due to ceiling effects.

|  | Estimate | SE | 95% CI | χ² | *df* | *p*(χ²) | *z-*Value | *p-*Value |
| --- | --- | --- | --- | --- | --- | --- | --- | --- |
| Intercept | 2.34 | 0.60 | [1.31, 3.76] |  |  |  | 3.86 | < .001*** |
| Country |  |  |  | 21.09 | 4 | < .001 *** |  |  |
| *Madagascar* | -0.83 | 0.75 | [-2.46, 0.60] |  |  |  | -1.10 | .271 |
| *Mongolia* | 0.44 | 0.95 | [-1.42, 2.51] |  |  |  | 0.46 | .644 |
| *Peru* | -2.20 | 0.71 | [-3.77, -0.92] |  |  |  | -3.12 | .002** |
| *Vanuatu* | -1.49 | 0.72 | [-3.08, -0.15] |  |  |  | -2.06 | .040* |

**Table S9.** **Binary GLM for questionnaire variable”Owner Feelings”**.

* *p* ≤ .05, ** *p* ≤ .01, ***  *p* ≤ .001.

| Contrast | Estimate | SE | *z*-Ratio | *p*-Value |
| --- | --- | --- | --- | --- |
| Germany – Madagascar | 0.831 | 0.755 | 1.102 | .806 |
| Germany – Mongolia | -0.437 | 0.947 | -0.462 | .991 |
| Germany – Peru | 2.202 | 0.707 | 3.115 | .016* |
| Germany – Vanuatu | 1.488 | 0.724 | 2.055 | .240 |
| Madagascar – Mongolia | -1.269 | 0.857 | -1.480 | .576 |
| Madagascar – Peru | 1.371 | 0.581 | 2.359 | .127 |
| Madagascar – Vanuatu | 0.657 | 0.602 | 1.091 | .811 |
| Mongolia – Peru | 2.639 | 0.816 | 3.236 | .011* |
| Mongolia – Vanuatu | 1.925 | 0.831 | 2.318 | .139 |
| Peru – Vanuatu | -0.714 | 0.541 | -1.319 | .679 |

**Table S10. Pairwise Comparison between Countries on Questionnaire Variable: “Owner Feelings”.**

**Results are presented on the log odds ratio scale. *p*-Values were adjusted using the Tukey method for comparing a family of five estimates. * *p* ≤ .05, ** *p* ≤ .01, *** *p* ≤ .001.**

|  | Estimate | SE | 95% CI | χ² | *p-*Value |
| --- | --- | --- | --- | --- | --- |
| Intercept | -1.68 | 0.46 | [-2.70, -0.85] | 18.08 | <.001*** |
| Country |  |  |  | 40.55 | < .001*** |
| *Madagascar* | 0.24 | 0.64 | [-1.02, 1.53] | 0.14 | .709 |
| *Mongolia* | -1.36 | 0.96 | [-3.68, 0.33] | 2.44 | .119 |
| *Peru* | 2.07 | 0.59 | [0.97, 3.31] | 14.27 | <.001*** |
| *Vanuatu* | -2.43 | 1.50 | [-7.33, -0.18] | 4.65 | .031* |

**Table S11.** **Firth's Logistic Regression for Questionnaire Variable: “Owner Regrets”.**

Usage of Firth's Logistic Regression due to complete separation issues.* *p* ≤ .05, ** *p* ≤ .01, *** *p* ≤ .001.

| Contrast | Estimate | SE | *t*-Ratio | *p*-Value |
| --- | --- | --- | --- | --- |
| Germany – Madagascar | -0.237 | 0.637 | -0.372 | .996 |
| Germany – Mongolia | 1.365 | 0.956 | 1.428 | .611 |
| Germany – Peru | -2.072 | 0.591 | -3.503 | .005** |
| Germany – Vanuatu | 2.431 | 1.500 | 1.621 | .486 |
| Madagascar – Mongolia | 1.602 | 0.943 | 1.700 | .437 |
| Madagascar – Peru | -1.834 | 0.569 | -3.221 | .013* |
| Madagascar – Vanuatu | 2.668 | 1.490 | 1.790 | .383 |
| Mongolia – Peru | -3.437 | 0.912 | -3.767 | .002** |
| Mongolia – Vanuatu | 1.066 | 1.650 | 0.645 | .967 |
| Peru – Vanuatu | 4.503 | 1.470 | 3.059 | .022* |

**Table S12.** **Pairwise Comparison between Countries on Questionnaire Variable: “Owner Regrets”.**

**Results are presented on the log odds ratio scale. df = 154. *p*-Values were adjusted using the Tukey method for comparing a family of five estimates. * *p* ≤ .05, ** *p* ≤ .01, *** *p* ≤ .001.**

|  | Estimate | SE | 95% CI | χ² | *df* | *p*(χ²) | *z-*Value | *p-*Value |
| --- | --- | --- | --- | --- | --- | --- | --- | --- |
| Intercept | 1.22 | 0.09 | [1.05, 1.39] |  |  |  | 13.99 | <.001*** |
| Country |  |  |  | 22.97 | 4 | < .001*** |  |  |
| *Madagascar* | 0.06 | 0.12 | [-0.18, 0.30] |  |  |  | 0.48 | .631 |
| *Mongolia* | -0.03 | 0.12 | [-0.27, 0.21] |  |  |  | -0.25 | .802 |
| *Peru* | -0.39 | 0.12 | [-0.63, -0.16] |  |  |  | -3.26 | .001** |
| *Vanuatu* | 0.21 | 0.13 | [-0.05, 0.47] |  |  |  | 1.61 | .107 |

**Table S13.** **Beta GLM for Relationship Quality Score.**

***** *p* ≤ .05, ** *p* ≤ .01, *** *p* ≤ .001.

| Contrast | Estimate | SE | *z-Ratio* | *p*-Value |
| --- | --- | --- | --- | --- |
| Germany – Madagascar | -0.01 | 0.02 | -0.48 | .989 |
| Germany – Mongolia | 0.01 | 0.02 | 0.25 | .999 |
| Germany – Peru | 0.08 | 0.02 | 3.27 | .010* |
| Germany – Vanuatu | -0.03 | 0.02 | -1.62 | .486 |
| Madagascar – Mongolia | 0.02 | 0.02 | 0.73 | .951 |
| Madagascar – Peru | 0.09 | 0.02 | 3.72 | .002** |
| Madagascar – Vanuatu | -0.02 | 0.02 | -1.14 | .786 |
| Mongolia – Peru | 0.07 | 0.02 | 3.00 | .023* |
| Mongolia – Vanuatu | -0.04 | 0.02 | -1.85 | .345 |
| Peru – Vanuatu | -0.11 | 0.02 | -4.77 | <.001*** |

**Table S14.** **Pairwise Comparison between Countries on Relationship Quality Score.**

*p*-Values were adjusted using the Tukey method for comparing a family of five estimates. * *p* ≤ .05, ** *p* ≤ .01, *** *p* ≤ .001.

**Additional Results: Obedience**

| Country | Successful Approach ≤ 20s | Approach Latency |
| --- | --- | --- |
| Germany | 1.00 (0.00) | 2.67 (0.53) |
| Madagascar | 0.66 (0.09) | 5.86 (0.82) |
| Mongolia | 0.50 (0.09) | 4.85 (0.78) |
| Peru | 0.52 (0.09) | 6.25 (0.82) |
| Vanuatu | 0.56 (0.10) | 8.57 (1.14) |

**Table S15.** **Descriptive Values for Obedience Test by country.**

Values are presented as means and standard errors (*M* (*SE*)).

|  | Estimate | SE | 95% CI | χ² | *p-*Value |
| --- | --- | --- | --- | --- | --- |
| Intercept | 3.84 | 1.42 | [1.87, 8.68] | 28.25 | <.001*** |
| Country |  |  |  | 20.48 | <.001*** |
| *Madagascar* | -3.25 | 1.47 | [-8.13, -1.11] | 11.41 | .001** |
| *Mongolia* | -3.78 | 1.48 | [-8.66, -1.62] | 16.49 | <.001*** |
| *Peru* | -3.54 | 1.48 | [-8.42, -1.38] | 13.83 | <.001*** |
| *Vanuatu* | -3.71 | 1.48 | [-8.60, -1.54] | 15.53 | <.001*** |
| Relationship Quality Score (z*)* | 0.28 | 0.21 | [-0.12, 0.70] | 1.91 | .167 |

**Table S16.** **Firth's Logistic Regression for Obedience Test: Successful Approach ≤ 20s.**

Usage of Firth's Logistic Regression due to complete separation issues.* *p* ≤ .05, ** *p* ≤ .01, *** *p* ≤ .001.

| Contrast | Estimate | SE | *t*-Ratio | *p*-Value |
| --- | --- | --- | --- | --- |
| Madagascar – Germany | 3.254 | 1.470 | 2.215 | .181 |
| Mongolia – Germany | 3.777 | 1.480 | 2.559 | .084 |
| Peru – Germany | 3.537 | 1.480 | 2.397 | .123 |
| Vanuatu – Germany | 3.713 | 1.480 | 2.512 | .094 |
| Mongolia – Madagascar | 0.523 | 0.538 | 0.971 | .868 |
| Peru – Madagascar | 0.282 | 0.536 | 0.526 | .984 |
| Vanuatu – Madagascar | 0.458 | 0.543 | 0.844 | .916 |
| Peru – Mongolia | -0.240 | 0.552 | -0.435 | .993 |
| Vanuatu – Mongolia | -0.064 | 0.562 | -0.114 | 1.000 |
| Vanuatu – Peru | 0.176 | 0.573 | 0.307 | .998 |

**Table S17.** **Pairwise Comparison between Countries on Obedience Test: Successful Approach ≤ 20s*.***

**Results are presented on the log odds ratio scale. df = 154. *p*-Values were adjusted using the Tukey method for comparing a family of five estimates. * *p* ≤ .05, ** *p* ≤ .01, *** *p* ≤ .001.**

|  | Estimate | SE | 95% CI | χ² | *df* | *p*(χ²) | *t-*Value | *p-*Value |
| --- | --- | --- | --- | --- | --- | --- | --- | --- |
| Intercept | 1.00 | 0.18 | [0.66, 1.39] |  |  |  | 5.49 | <.001*** |
| Country |  |  |  | 17.49 | 4 | .001** |  |  |
| *Madagascar* | 0.80 | 0.26 | [0.28, 1.33] |  |  |  | 3.02 | .003** |
| *Mongolia* | 0.58 | 0.31 | [0.00, 1.21] |  |  |  | 1.90 | .061 |
| *Peru* | 0.75 | 0.30 | [0.16, 1.39] |  |  |  | 2.58 | .012* |
| *Vanuatu* | 1.18 | 0.30 | [0.61, 1.79] |  |  |  | 3.94 | <.001*** |
| Relationship Quality Score (z) | -0.09 | 0.12 | [-0.33, 0.15] | 0.51 | 1 | .475 | -0.73 | .468 |

**Table S18. Gamma GLM for Approach Latency.**

* *p* ≤ .05, ** *p* ≤ .01, *** *p* ≤ .001.

| Contrast | Estimate | SE | z-Value | *p*-Value |
| --- | --- | --- | --- | --- |
| Madagascar – Germany | 0.800 | 0.264 | 3.019 | .021* |
| Mongolia – Germany | 0.584 | 0.307 | 1.902 | .314 |
| Peru – Germany | 0.763 | 0.296 | 2.578 | .074 |
| Vanuatu – Germany | 1.185 | 0.301 | 3.935 | <.001*** |
| Mongolia – Madagascar | -0.216 | 0.316 | -0.684 | .960 |
| Peru – Madagascar | -0.037 | 0.307 | -0.122 | 1.000 |
| Vanuatu – Madagascar | 0.385 | 0.307 | 1.252 | .718 |
| Peru – Mongolia | 0.179 | 0.333 | 0.536 | .983 |
| Vanuatu – Mongolia | 0.601 | 0.350 | 1.717 | .421 |
| Vanuatu – Peru | 0.422 | 0.345 | 1.222 | .736 |

**Table S19.** **Pairwise Comparison between Countries on Obedience Test: Approach Latency.**

*p*-Values were adjusted using the Tukey method for comparing a family of five estimates. * *p* ≤ .05, ** *p* ≤ .01, *** *p* ≤ .001.

**Additional Results: Pointing**

| Country | *N* | *M* (*SE*) |
| --- | --- | --- |
| Germany | 32 | 0.756 (0.039) |
| Madagascar | 19 | 0.674 (0.051) |
| Mongolia | 31 | 0.702 (0.039) |
| Peru | 8 | 0.679 (0.060) |
| Vanuatu | 22 | 0.586 (0.067) |

**Table S20.**

**Descriptive values of Pointing Test by country.**

Values are presented as means and standard errors (*M* (*SE*)) of the Variable Choice Performance.

|  | Estimate | SE | 95% CI | χ² | *df* | *p*(χ²) | *z*-Value | *p*-Value |
| --- | --- | --- | --- | --- | --- | --- | --- | --- |
| Intercept | 1.19 | 0.20 | [0.80, 1.61] |  |  |  | 6.06 | <.001*** |
| Country |  |  |  |  |  |  |  |  |
| *Madagascar* | -0.44 | 0.30 | [-1.04, 0.18] | 8.49 | 4 | .075 | -1.44 | .151 |
| *Mongolia* | -0.36 | 0.27 | [-0.91, 0.19] |  |  |  | -1.33 | .182 |
| *Peru* | -0.43 | 0.45 | [-1.26, 0.60] |  |  |  | -0.96 | .336 |
| *Vanuatu* | -0.87 | 0.30 | [-1.44, -0.29] |  |  |  | -2.92 | .004** |
| Trial (z) | -0.10 | 0.10 | [-0.31, 0.08] | 1.20 | 1 | .273 | -1.09 | .274 |
| Relationship Quality Score (z) | 0.06 | 0.11 | [-0.16, 0.28] | 0.29 | 1 | .591 | 0.54 | .591 |

**Table S21.** **GLMM results for Pointing Test: Choice Performance.**

* *p* ≤ .05, ** *p* ≤ .01, *** *p* ≤ .001.

**Additional Results: Showing**

| Country | Owner's choice | Dog's first look |
| --- | --- | --- |
| Germany | 0.56 (0.06) | 0.38 (0.06) |
| Madagascar | 0.65 (0.08) | 0.26 (0.07) |
| Mongolia | 0.48 (0.06) | 0.29 (0.05) |
| Peru | 0.43 (0.07) | 0.22 (0.06) |
| Vanuatu | 0.82 (0.04) | 0.34 (0.06) |

**Table S22.** **Descriptive values of Showing Test by country.**

Values are presented as means and standard errors (*M* (*SE*)).

|  | Estimate | SE | 95% CI | χ² | *df* | *p*(χ²) | *z-*Value | *p*-Value |
| --- | --- | --- | --- | --- | --- | --- | --- | --- |
| Intercept | 0.25 | 0.26 | [-0.30, 0.79] |  |  |  | 0.99 | .322 |
| Country |  |  |  | 17.56 | 4 | .002** |  |  |
| *Madagascar* | 0.31 | 0.39 | [-0.44, 1.16] |  |  |  | 0.79 | .431 |
| *Mongolia* | -0.33 | 0.37 | [-1.12, 0.44] |  |  |  | -0.89 | .373 |
| *Peru* | -0.37 | 0.39 | [-1.24, 0.48] |  |  |  | -0.95 | .342 |
| *Vanuatu* | 1.19 | 0.42 | [0.44, 2.30] |  |  |  | 2.80 | .005** |
| Trial (z) | 0.12 | 0.13 | [-0.13, 0.37] | 0.96 | 1 | .326 | 0.98 | .327 |
| Relationship Quality Score (z) | 0.05 | 0.15 | [-0.29, 0.36] | 0.11 | 1 | .738 | 0.33 | .738 |

**Table S23.** **GLMM results for Showing Test: Owner's choice.**

* *p* ≤ .05, ** *p* ≤ .01, *** *p* ≤ .001.

| Contrast | Estimate | SE | *z*-Value | *p*-Value |
| --- | --- | --- | --- | --- |
| Madagascar – Germany | 0.31 | 0.39 | 0.79 | .934 |
| Mongolia – Germany | -0.33 | 0.37 | -0.89 | .900 |
| Peru – Germany | -0.37 | 0.39 | -0.95 | .876 |
| Vanuatu – Germany | 1.19 | 0.42 | 2.80 | .041* |
| Mongolia – Madagascar | -0.63 | 0.40 | -1.60 | .493 |
| Peru – Madagascar | -0.68 | 0.42 | -1.60 | .492 |
| Vanuatu – Madagascar | 0.88 | 0.45 | 1.96 | .284 |
| Peru – Mongolia | -0.05 | 0.39 | -0.12 | .999 |
| Vanuatu – Mongolia | 1.51 | 0.43 | 3.54 | .004** |
| Vanuatu – Peru | 1.56 | 0.46 | 3.42 | .006** |

**Table S24.** **Pairwise Comparison between countries for Showing Test: Owner's choice.**

*z-*Values are Tukey-adjusted *z* statistics. *p*-Values were adjusted using the Tukey method for comparing a family of five estimates. * *p* ≤ .05, ** *p* ≤ .01, *** *p* ≤ .001.

|  | Estimate | SE | 95% CI | χ² | *df* | *p*(χ²) | *z-*Value | *p*-Value |
| --- | --- | --- | --- | --- | --- | --- | --- | --- |
| Intercept | -0.51 | 0.26 | [–1.11, –0.04] |  |  |  | -1.96 | .050 |
| Country |  |  |  | 4.39 | 4 | .356 |  |  |
| *Madagascar* | -0.55 | 0.42 | [-1.51, 0.28] |  |  |  | -1.31 | .189 |
| *Mongolia* | -0.49 | 0.43 | [-1.45, 0.31] |  |  |  | -1.16 | .248 |
| *Peru* | -0.81 | 0.44 | [-1.85, -0.01] |  |  |  | -1.83 | .067 |
| *Vanuatu* | -0.15 | 0.43 | [-1.07, 0.72] |  |  |  | -0.35 | .723 |
| Trial (z) | -0.18 | 0.14 | [-0.48, 0.09] | 1.64 | 1 | .200 | -1.28 | .202 |
| Relationship Quality Score (z) | 0.00 | 0.17 | [-0.36, 0.34] | 0.00 | 1 | .984 | -0.02 | .984 |

**Table S25.** **GLMM results for Showing Test: Dog's first look.**

* *p* ≤ .05, ** *p* ≤ .01, *** *p* ≤ .001.

**Additional results: Perspective Taking Test**

| Country | *N* | Food taken | Duration | Dog looked at Owner |
| --- | --- | --- | --- | --- |
| Germany | 31 | 0.36 (0.08) | 46.2 (3.38) | 0.92 (0.04) |
| Madagascar | 20 | 0.40 (0.10) | 44.2 (4.65) | 0.90 (0.06) |
| Mongolia | 7 | 0.71 (0.09) | 19.1 (5.02) | 0.43 (0.10) |
| Peru | 9 | 0.56 (0.11) | 33.7 (6.23) | 0.67 (0.11) |
| Vanuatu | 16 | 0.94 (0.05) | 17.6 (3.48) | 0.50 (0.09) |

**Table S26.** **Descriptive Values of Perspective Taking Test (Eyes closed condition) by country.**

Values are presented as means and standard errors (*M* (*SE*)).

| Country | N | Food taken | Duration | Dog looked at Owner |
| --- | --- | --- | --- | --- |
| Germany | 32 | 0.30 (0.08) | 42.7 (3.70) | 0.84 (0.06) |
| Madagascar | 16 | 0.50 (0.10) | 35.6 (5.17) | 0.88 (0.07) |
| Mongolia | 7 | 0.71 (0.09) | 18.9 (5.07) | 0.29 (0.09) |
| Peru | 10 | 0.60 (0.11) | 31.7 (6.19) | 0.90 (0.07) |
| Vanuatu | 17 | 0.77 (0.08) | 20.2 (4.27) | 0.41 (0.09) |

**Table S27.** **Descriptive values of Perspective Taking Test (Eyes open condition) by country.**

Values are presented as means and standard errors (*M* (*SE*)).

|  | Estimate | SE | 95% CI | χ² | *df* | *p*(χ²) | *z-*Value | *p-*Value |
| --- | --- | --- | --- | --- | --- | --- | --- | --- |
| Intercept | -1.00 | 0.80 | [-13.79, 0.31] |  |  |  |  | .208 |
| Condition (eyes closed vs. open) | -0.15 | 0.53 | [-2.70, 1.79] | 0.08 | 1 | .778 | -0.28 | .779 |
| Country |  |  |  | 17.97 | 4 | .001** |  |  |
| *Madagascar* | 0.44 | 1.09 | [-1.84, 13.90] | 1 |  |  | 0.40 | .686 |
| *Mongolia* | 3.23 | 2.01 | [0.47, 29.62] |  |  |  | 1.61 | .108 |
| *Peru* | 1.70 | 1.54 | [-1.17, 25.86] |  |  |  | 1.11 | .268 |
| *Vanuatu* | 4.38 | 1.68 | [1.90, 29.90] |  |  |  | 2.61 | .009** |
| Trial (z) | -0.38 | 0.28 | [-4.56, 0.15] | 2.14 | 1 | .143 | -1.38 | .166 |
| Relationship Quality Score (z) | -0.29 | 0.43 | [-1.68, 0.54] | 0.45 | 1 | .503 | -0.66 | .507 |

**Table S28.** **GLMM results for Perspective Taking Test: Food Taken.**

* *p* ≤ .05, ** *p* ≤ .01, *** *p* ≤ .001.

| Contrast | Estimate | SE | *z*-Value | *p*-Value |
| --- | --- | --- | --- | --- |
| Madagascar – Germany | 0.44 | 1.09 | 0.40 | .994 |
| Mongolia – Germany | 3.23 | 2.01 | 1.61 | .478 |
| Peru – Germany | 1.70 | 1.54 | 1.11 | .794 |
| Vanuatu – Germany | 4.38 | 1.68 | 2.61 | .065 |
| Mongolia – Madagascar | 2.79 | 2.03 | 1.37 | .632 |
| Peru – Madagascar | 1.26 | 1.59 | 0.79 | .929 |
| Vanuatu – Madagascar | 3.93 | 1.70 | 2.31 | .133 |
| Peru – Mongolia | -1.53 | 1.96 | -0.78 | .933 |
| Vanuatu – Mongolia | 1.14 | 1.73 | 0.66 | .962 |
| Vanuatu – Peru | 2.67 | 1.63 | 1.64 | .461 |

**Table S29.** **Pairwise comparison between countries for Perspective Taking Test: Food Taken.**

*z-*Values are Tukey-adjusted *z* statistics. *p*-Values were adjusted using the Tukey method for comparing a family of five estimates. * *p* ≤ .05, ** *p* ≤ .01, *** *p* ≤ .001.

|  | Estimate | SE | 95% CI | χ² | *df* | *p*(χ²) | *z-*Value | *p-*Value |
| --- | --- | --- | --- | --- | --- | --- | --- | --- |
| Intercept | 3.49 | 1.55 | [1.98, 14.61] |  |  |  | 2.26 | .024* |
| Condition (closed vs. open) | 0.66 | 0.62 | [-0.81, 8.74] | 1.33 | 1 | .248 | 1.07 | .283 |
| Country |  |  |  | 22.55 | 4 | <.001*** |  |  |
| *Madagascar* | 0.01 | 1.13 | [-2.44, 15.55] |  |  |  | 0.01 | .996 |
| *Mongolia* | -5.26 | 2.67 | [-30.04, -2.25] |  |  |  | -1.97 | .049* |
| *Peru* | -1.52 | 1.38 | [-7.28, 15.16] |  |  |  | -1.10 | .273 |
| *Vanuatu* | -4.22 | 2.02 | [-26.36, -1.97] |  |  |  | -2.09 | .037* |
| Trial (z) | -0.15 | 0.29 | [-2.67, 1.25] | 0.26 | 1 | .608 | -0.50 | .616 |
| Relationship Quality Score (z) | -0.59 | 0.49 | [-3.81, 0.30] | 1.91 | 1 | .167 | -1.20 | .229 |

**Table S30.** **GLMM results for Perspective Taking Test: Dog looked at owner.**

* *p* ≤ .05, ** *p* ≤ .01, *** *p* ≤ .001.

| Contrast | Estimate | SE | *z*-Value | *p*-Value |
| --- | --- | --- | --- | --- |
| Madagascar – Germany | 0.01 | 1.13 | 0.01 | .999 |
| Mongolia – Germany | -5.26 | 2.67 | -1.97 | .251 |
| Peru – Germany | -1.52 | 1.38 | -1.10 | .781 |
| Vanuatu – Germany | -4.22 | 2.02 | -2.09 | .197 |
| Mongolia – Madagascar | -5.26 | 2.83 | -1.86 | .303 |
| Peru – Madagascar | -1.52 | 1.54 | -0.99 | .838 |
| Vanuatu – Madagascar | -4.22 | 2.19 | -1.93 | .269 |
| Peru – Mongolia | 3.74 | 2.52 | 1.48 | .534 |
| Vanuatu – Mongolia | 1.04 | 1.59 | 0.65 | .960 |
| Vanuatu – Peru | -2.70 | 1.93 | -1.40 | .592 |

**Table S31.** **Pairwise comparison between countries for Perspective Taking Test: Dog looked at owner.**

*z-*Values are Tukey-adjusted *z* statistics. *p*-Values were adjusted using the Tukey method for comparing a family of five estimates. * *p* ≤ .05, ** *p* ≤ .01, *** *p* ≤ .001.

**Additional results: Unsolvable task**

| Country | First Look to Owner | First Look to Experimenter | Persistence |
| --- | --- | --- | --- |
| Germany | 0.47 (0.09) | 0.31 (0.08) | 0.54 (0.06) |
| Madagascar | 0.20 (0.07) | 0.80 (0.07) | 0.19 (0.02) |
| Mongolia | 0.13 (0.06) | 0.60 (0.08) | 0.35 (0.04) |
| Peru | 0.19 (0.07) | 0.63 (0.09) | 0.19 (0.02) |
| Vanuatu | 0.10 (0.06) | 0.84 (0.07) | 0.18 (0.02) |

**Table S32.** **Descriptive values of Unsolvable Task by country.**

Values are presented as means and standard errors (*M* (*SE*)).

|  | Estimate | SE | 95% CI | χ² | *df* | *p*(χ²) | *z*-Value | *p-*Value |
| --- | --- | --- | --- | --- | --- | --- | --- | --- |
| Intercept | -0.14 | 0.36 | [-0.85, 0.56] |  |  |  | -0.39 | .693 |
| Country |  |  |  | 12.88 | 4 | .012* |  |  |
| *Madagascar* | -1.27 | 0.61 | [-2.56, -0.12] |  |  |  | -2.07 | .038* |
| *Mongolia* | -1.69 | 0.65 | [-3.08, -0.50] |  |  |  | -2.63 | .009** |
| *Peru* | -1.25 | 0.65 | [-2.60, -0.02] |  |  |  | -1.92 | .054 |
| *Vanuatu* | -2.10 | 0.83 | [-4.05, -0.65] |  |  |  | -2.53 | .011* |
| Relationship Quality Score (z) | 0.12 | 0.26 | [-0.38, 0.64] | 0.22 | 1 | .641 | 0.46 | .643 |

**Table S33.** **GLMM results for Unsolvable Task: First Look to Owner.**

* *p* ≤ .05, ** *p* ≤ .01, *** *p* ≤ .001.

| Contrast | Estimate | SE | *z*-Value | *p*-Value |
| --- | --- | --- | --- | --- |
| Madagascar – Germany | -1.27 | 0.61 | -2.07 | .226 |
| Mongolia – Germany | -1.69 | 0.65 | -2.63 | .063 |
| Peru – Germany | -1.25 | 0.65 | -1.92 | .297 |
| Vanuatu – Germany | -2.10 | 0.83 | -2.53 | .080 |
| Mongolia – Madagascar | -0.42 | 0.74 | -0.57 | .978 |
| Peru – Madagascar | 0.02 | 0.75 | 0.03 | 1.000 |
| Vanuatu – Madagascar | -0.83 | 0.90 | -0.92 | .885 |
| Peru – Mongolia | 0.44 | 0.76 | 0.59 | .976 |
| Vanuatu – Mongolia | -0.41 | 0.92 | -0.44 | .992 |
| Vanuatu – Peru | -0.85 | 0.94 | -0.90 | .893 |

**Table S34.** **Pairwise comparison between countries for Unsolvable Task: First Look to Owner.**

*z-*Values are Tukey-adjusted *z* statistics. *p*-Values were adjusted using the Tukey method for comparing a family of five estimates. * *p* ≤ .05, ** *p* ≤ .01, *** *p* ≤ .001.

|  | Estimate | SE | 95% CI | χ² | *df* | *p*(χ²) | *z*-Value | *p-*Value |
| --- | --- | --- | --- | --- | --- | --- | --- | --- |
| Intercept | -0.79 | 0.38 | [-1.58, -0.06] |  |  |  | -2.05 | .040* |
| Country |  |  |  | 20.74 | 4 | <.001*** |  |  |
| *Madagascar* | 2.18 | 0.63 | [1.00, 3.50] |  |  |  | 3.46 | <.001*** |
| *Mongolia* | 1.13 | 0.54 | [0.10, 2.22] |  |  |  | 2.11 | .035* |
| *Peru* | 1.30 | 0.59 | [0.17, 2.49] |  |  |  | 2.21 | .027* |
| *Vanuatu* | 2.47 | 0.74 | [1.13, 4.09] |  |  |  | 3.35 | <.001*** |
| Relationship Quality Score (z) | -0.03 | 0.22 | [-0.46, 0.41] | 0.01 | 1 | .909 | -0.11 | .909 |

**Table S35.** **GLMM results for Unsolvable Task: First Look to Experimenter.**

* *p* ≤ .05, ** *p* ≤ .01, *** *p* ≤ .001.

| Contrast | Estimate | SE | *z*-Value | *p*-Value |
| --- | --- | --- | --- | --- |
| Madagascar – Germany | 2.18 | 0.63 | 3.46 | .005** |
| Mongolia – Germany | 1.13 | 0.54 | 2.11 | .212 |
| Peru – Germany | 1.30 | 0.59 | 2.21 | .174 |
| Vanuatu – Germany | 2.47 | 0.74 | 3.35 | .007** |
| Mongolia – Madagascar | -1.04 | 0.63 | -1.66 | .452 |
| Peru – Madagascar | -0.88 | 0.68 | -1.30 | .685 |
| Vanuatu – Madagascar | 0.29 | 0.80 | 0.36 | .996 |
| Peru – Mongolia | 0.16 | 0.58 | 0.28 | .999 |
| Vanuatu – Mongolia | 1.33 | 0.74 | 1.81 | .363 |
| Vanuatu – Peru | 1.17 | 0.79 | 1.49 | .562 |

**Table S36.** **Pairwise comparison between countries for Unsolvable Task: First Look to Experimenter.**

***z-*Values are Tukey-adjusted *z* statistics. *p*-Values were adjusted using the Tukey method for comparing a family of five estimates. * *p* ≤ .05, ** *p* ≤ .01, *** *p* ≤ .001.**

|  | Estimate | SE | 95% CI | χ² | *df* | *p*(χ²) | *z*-Value | *p-*Value |
| --- | --- | --- | --- | --- | --- | --- | --- | --- |
| Intercept | 0.30 | 0.18 | [-0.06, 0.65] |  |  |  | 1.65 | .100 |
| Country |  |  |  | 35.33 | 4 | <.001*** |  |  |
| *Madagascar* | -1.34 | 0.28 | [-1.88, -0.80] |  |  |  | -4.86 | <.001*** |
| *Mongolia* | -0.64 | 0.26 | [-1.15, -0.12] |  |  |  | -2.44 | .015* |
| *Peru* | -1.30 | 0.28 | [-1.85, -0.75] |  |  |  | -4.63 | <.001*** |
| *Vanuatu* | -1.50 | 0.30 | [-2.10, -0.90] |  |  |  | -4.92 | <.001*** |
| Relationship Quality Score (z) | 0.08 | 0.10 | [-0.11, 0.28] | 0.63 | 1 | 0.439 | 0.82 | .413 |

**Table S37.** **Beta GLM results for Unsolvable Task: Persistence*.***

** p ≤* .05, ** *p ≤* .01*, *** p ≤* .001.

| Contrast | Estimate | SE | *z*-Ratio | *p*-Value |
| --- | --- | --- | --- | --- |
| Germany – Madagascar | 0.31 | 0.06 | 5.25 | <.001*** |
| Germany – Mongolia | 0.16 | 0.06 | 2.48 | .096 |
| Germany – Peru | 0.31 | 0.06 | 4.98 | <.001*** |
| Germany – Vanuatu | 0.34 | 0.06 | 5.52 | <.001*** |
| Madagascar – Mongolia | -0.16 | 0.06 | -2.57 | .075 |
| Madagascar – Peru | -0.01 | 0.06 | -0.14 | 1.000 |
| Madagascar – Vanuatu | 0.03 | 0.06 | 0.50 | .987 |
| Mongolia – Peru | 0.15 | 0.06 | 2.41 | .111 |
| Mongolia – Vanuatu | 0.18 | 0.06 | 2.94 | .027* |
| Peru – Vanuatu | 0.04 | 0.06 | 0.62 | .973 |

**Table S38.** **Pairwise comparison between countries for Unsolvable Task: Persistence**.

*z-*Values are Tukey-adjusted *z* statistics. *p*-Values were adjusted using the Tukey method for comparing a family of five estimates. * *p* ≤ .05, ** *p* ≤ .01, *** *p* ≤ .001.

**Additional results: Social Referencing Test**

| Country | Approach with Owner | Look at Owner | Joined Play |
| --- | --- | --- | --- |
| Germany | 0.90 (0.05) | 0.90 (0.05) | 0.78 (0.07) |
| Madagascar | 0.55 (0.09) | 1.00 (0.00) | 0.13 (0.06) |
| Mongolia | 0.53 (0.09) | 0.93 (0.04) | 0.03 (0.03) |
| Peru | 0.60 (0.09) | 0.93 (0.04) | 0.10 (0.05) |
| Vanuatu | 0.40 (0.09) | 1.00 (0.00) | 0.00 (0.00) |

**Table S39.** **Descriptive values for Social Referencing by country.**

Values are presented as means and standard errors (*M* (*SE*)).

|  | Estimate | SE | 95% CI | χ² | *df* | *p*(χ²) | *z*-Value | *p-*Value |
| --- | --- | --- | --- | --- | --- | --- | --- | --- |
| Intercept | 2.18 | 0.61 | [1.12, 3.62] |  |  |  | 3.54 | <.001*** |
| Country |  |  |  | 22.65 | 4 | <.001*** |  |  |
| *Madagascar* | -2.13 | 0.73 | [-3.73, -0.81] |  |  |  | -2.93 | 0.003** |
| *Mongolia* | -2.00 | 0.73 | [-3.60, -0.68] |  |  |  | -2.76 | 0.006** |
| *Peru* | -1.32 | 0.74 | [-2.94, 0.05] |  |  |  | -1.77 | 0.076 |
| *Vanuatu* | -2.89 | 0.74 | [-4.51, -1.55] |  |  |  | -3.91 | <.001*** |
| Relationship Quality Score (z) | 0.71 | 0.23 | [0.28, 1.20] | 10.70 | 1 | 0.001** | 3.04 | 0.002** |

**Table S40.** **GLM results for Social Referencing: Approach with Owner.**

* *p* ≤ .05, ** *p* ≤ .01, *** *p* ≤ .001.

| Contrast | Estimate | SE | *z*-Value | *p*-Value |
| --- | --- | --- | --- | --- |
| Madagascar - Germany | -2.13 | 0.73 | -2.93 | 0.027* |
| Mongolia - Germany | -2.00 | 0.73 | -2.76 | 0.045* |
| Peru - Germany | -1.32 | 0.74 | -1.77 | 0.384 |
| Vanuatu - Germany | -2.89 | 0.74 | -3.91 | <0.001*** |
| Mongolia - Madagascar | 0.12 | 0.54 | 0.23 | 0.999 |
| Peru - Madagascar | 0.81 | 0.59 | 1.38 | 0.639 |
| Vanuatu - Madagascar | -0.76 | 0.55 | -1.39 | 0.628 |
| Peru - Mongolia | 0.69 | 0.58 | 1.19 | 0.754 |
| Vanuatu - Mongolia | -0.88 | 0.56 | -1.58 | 0.503 |
| Vanuatu - Peru | -1.57 | 0.62 | -2.54 | 0.079 |

**Table S41.** **Pairwise comparison between countries for Social Referencing: Approach with Owner.**

*z-*Values are Tukey-adjusted *z* statistics. *p*-Values were adjusted using the Tukey method for comparing a family of five estimates. * *p* ≤ .05, ** *p* ≤ .01, *** *p* ≤ .001.

|  | Estimate | SE | 95% CI | χ² | *p-*Value |
| --- | --- | --- | --- | --- | --- |
| Intercept | 2.09 | 0.57 | [1.09, 3.42] | 21.15 | <0.001*** |
| Country |  |  |  | 4.78 | .311 |
| *Madagascar* | 2.08 | 1.49 | [-0.32, 7.00] | 2.76 | .097 |
| *Mongolia* | 0.30 | 0.86 | [-1.41, 2.16] | 0.12 | .727 |
| *Peru* | 0.13 | 0.89 | [-1.66, 2.09] | 0.02 | .883 |
| *Vanuatu* | 2.07 | 1.50 | [-0.35, 7.00] | 2.68 | .101 |
| Relationship Quality Score (z) | -0.30 | 0.44 | [-1.38, 0.69] | 0.33 | .565 |

**Table S42.** **Firth's logistic regression for Social Referencing: Look at Owner.**

Usage of Firth's Logistic Regression due to complete separation issues.* *p* ≤ .05, ** *p* ≤ .01, *** *p* ≤ .001.

|  | Estimate | SE | 95% CI | χ² | *p-*Value |
| --- | --- | --- | --- | --- | --- |
| Intercept | 1.13 | 0.42 | [0.35, 2.02] | 8.36 | .004** |
| Country |  |  |  | 67.10 | <.001*** |
| *Madagascar* | -3.11 | 0.68 | [-4.59, -1.86] | 27.99 | <.001*** |
| *Mongolia* | -4.19 | 0.94 | [-6.51, -2.59] | 38.02 | <.001*** |
| *Peru* | -2.92 | 0.71 | [-4.46, -1.62] | 21.50 | <.001*** |
| *Vanuatu* | -5.59 | 1.49 | [-10.50, -3.35] | 47.85 | <.001*** |
| Relationship Quality Score (z) | 0.64 | 0.37 | [-0.10, 1.46] | 2.85 | .091 |

**Table S43.** **Firth's Logistic Regression for Social Referencing: Joined Play.**

Usage of Firth's Logistic Regression due to complete separation issues.* *p* ≤ .05, ** *p* ≤ .01, *** *p* ≤ .001.

| Contrast | Estimate | SE | *t*-Ratio | *p*-Value |
| --- | --- | --- | --- | --- |
| Germany – Madagascar | 3.12 | 0.68 | 4.58 | <.001*** |
| Germany – Mongolia | 4.19 | 0.94 | 4.47 | <.001*** |
| Germany – Peru | 2.92 | 0.71 | 4.13 | <.001*** |
| Germany – Vanuatu | 5.59 | 1.49 | 3.76 | .002** |
| Madagascar – Mongolia | 1.07 | 0.98 | 1.10 | .809 |
| Madagascar – Peru | –0.19 | 0.80 | –0.24 | .999 |
| Madagascar – Vanuatu | 2.47 | 1.49 | 1.65 | .467 |
| Mongolia – Peru | –1.26 | 1.02 | –1.24 | .730 |
| Mongolia – Vanuatu | 1.40 | 1.64 | 0.86 | .913 |
| Peru – Vanuatu | 2.66 | 1.55 | 1.72 | .427 |

**Table S44.** **Pairwise Comparison between Countries on Social Referencing: Joined Play**.

Results are presented on the log odds ratio scale. df = 154. *p*-Values were adjusted using the Tukey method for comparing a family of five estimates. * *p* ≤ .05, ** *p* ≤ .01, *** *p* ≤ .001.
